# Development of a Phage Cocktail to Control *Proteus mirabilis* Catheter-associated Urinary Tract Infections

**DOI:** 10.3389/fmicb.2016.01024

**Published:** 2016-06-28

**Authors:** Luís D. R. Melo, Patrícia Veiga, Nuno Cerca, Andrew M. Kropinski, Carina Almeida, Joana Azeredo, Sanna Sillankorva

**Affiliations:** ^1^Laboratório de Investigação em Biofilmes Rosário Oliveira, Centre of Biological Engineering, University of Minho BragaBraga, Portugal; ^2^Departments of Food Science, Molecular and Cellular Biology, and Pathobiology, University of Guelph, GuelphON, Canada

**Keywords:** bacteriophages, bacteriophage therapy, biofilms, urinary tract infection, *Proteus mirabilis*, phage cocktail

## Abstract

*Proteus mirabilis* is an enterobacterium that causes catheter-associated urinary tract infections (CAUTIs) due to its ability to colonize and form crystalline biofilms on the catheters surface. CAUTIs are very difficult to treat, since biofilm structures are highly tolerant to antibiotics. Phages have been used widely to control a diversity of bacterial species, however, a limited number of phages for *P. mirabilis* have been isolated and studied. Here we report the isolation of two novel virulent phages, the podovirus vB_PmiP_5460 and the myovirus vB_PmiM_5461, which are able to target, respectively, 16 of the 26 and all the *Proteus* strains tested in this study. Both phages have been characterized thoroughly and sequencing data revealed no traces of genes associated with lysogeny. To further evaluate the phages’ ability to prevent catheter’s colonization by *Proteus*, the phages adherence to silicone surfaces was assessed. Further tests in phage-coated catheters using a dynamic biofilm model simulating CAUTIs, have shown a significant reduction of *P. mirabilis* biofilm formation up to 168 h of catheterization. These results highlight the potential usefulness of the two isolated phages for the prevention of surface colonization by this bacterium.

## Introduction

Indwelling urinary catheters are medical devices used by millions of people to relieve urinary retention and urinary incontinence. It has been estimated that more than 100 million urethral catheters are fitted each year in a range of healthcare facilities (Getliffe and Newton, 2006). Despite the important benefits brought by the use of these devices, catheters provide a suitable surface for the colonization of microorganisms and may further place at risk the patients’ health, due to infections. In USA, catheter-associated urinary tract infections (CAUTIs) account for up to 40% of hospital-acquired infections (Saint et al., 2008). Also, 70% of urinary tract infections (UTIs) are associated with urinary catheters (Burton et al., 2011; Weber et al., 2011) and approximately 20% patients will suffer a catheterization during their hospital stay, especially in intensive care units (Saint and Lipsky, 1999).

The duration of the catheterization is a crucial risk factor of CAUTI development as almost all long-term catheterized (>28 days) patients develop a CAUTI, whereas in short-term (<7 days) catheterized patients only 10–50% develop an infection (Morris et al., 1999).

*Proteus mirabilis* is a leading cause of CAUTIs, being associated with up to 44% of all CAUTIs in the USA (O’Hara et al., 2000; Jacobsen et al., 2008). This microorganism, isolated from soil, stagnant water, sewage, and human intestinal tract, is associated with complicated infections, long-term catheterizations and urinary stone (struvite) formation (Armbruster and Mobley, 2012). *P. mirabilis* is a dimorphic bacterium that expresses thousands of flagella responsible for its swarming ability (Hoeniger, 1965). The virulence factors of *P. mirabilis* that contribute to the establishment of CAUTIs include the expression of fimbriae, that mediate the attachment to host cells and to catheters, and the consequent formation of dense biofilms on catheter surfaces (Armbruster and Mobley, 2012). Furthermore, it produces urease which is responsible for urea hydrolysis to carbon dioxide and ammonia that raises urine pH to above 8.3 (Stickler et al., 1998; Broomfield et al., 2009).

The microorganisms that colonize indwelling urinary catheters are commonly associated with antibiotic resistance and, thus, biofilm structures are frequently reported as reservoirs of antibiotic-resistant bacteria (Chenoweth et al., 2014). Although antibiotic therapy is successful in the majority of the cases, there has been a dramatic increase on antibiotic resistance among CAUTI-causing bacteria, including *P. mirabilis* (Wang et al., 2014). This fact makes it difficult to treat CAUTIs, highlighting the need for alternative preventive measures. During the last decades, the use of virulent bacteriophages (or phages) has re-emerged for therapeutic purposes (Viertel et al., 2014). After replicating inside the bacterial host, they cause cell lysis and release of phage progeny, which are able to infect neighboring cells. Phages are potential specific antibacterial agents, as they have self-replicating nature in the presence of the host cells, being eliminated from the human body in their absence (Azeredo and Sutherland, 2008). Furthermore, they are active against antibiotic-resistant bacteria, and phage preparations containing several phages, also known as phage cocktails, can be developed to increase their activity spectrum (Gu et al., 2012).

In the present study, two *P. mirabilis*-specific phages were isolated and characterized, and their effectiveness to control biofilm formation on silicone catheters was assessed in a dynamic biofilm model simulating CAUTIs.

## Materials and Methods

### Bacterial Strains and Culture Conditions

A total of 26 *Proteus* spp. (18 *P. mirabilis*) strains were used in this study. Additionally, other members of the *Enterobacteriaceae* were used to assess the lytic spectra of the phages. The complete list of strains used in this study is provided in **Table 1**. Reference strains were obtained from *Salmonella* Genetic Stock Centre (SGSC) and Colección Española de Cultivos Tipo (CECT), while isolates were obtained from Laboratório de Análises Clínicas S. Lázaro (Braga, Portugal), from urine samples. Species was identified using selective agar media and biochemical tests. Bacteria were grown at 37°C in Tryptic Soy Broth (TSB; Liofilchem), on Tryptic Soy Agar (TSA; 1.5% agar) or in Artificial Urine (AU) (Brooks and Keevil, 1997) supplemented with 0.3% glucose (Silva et al., 2010).

**Table 1:** Phages lytic spectra on the bacterial strains used in this study.

Species	Strain	Source	Infectivity Pm5460	Infectivity Pm5461
*Proteus mirabilis*	ATCC 29906^a^	Culture collection	+	+
	ATCC 14153^a^	Culture collection	+	+
	SGSC 5460^a^	Culture collection	+	+
	SGSC 5461^a^	Culture collection	-	+
	933^a^	Human urine isolate (Braga)	-	+
	SGSC 3360^a^	Culture collection	-	+
	SGSC 5446^a^	Culture collection	+	+
	SGSC 5447^a^	Culture collection	+	+
	SGSC 5445^a^	Culture collection	-	+
	SGSC 5448^a^	Culture collection	+	+
	SGSC 5449^a^	Culture collection	+	+
	SGSC 5450^a^	Culture collection	-	+
	2380	Human urine isolate (Braga)	+	+
	2388B	Human urine isolate (Braga)	+	+
	9229	Human urine isolate (Braga)	-	+
	SLaz1^a^	Human urine isolate (Braga)	+	+
	SLaz2	Human urine isolate (Braga)	+	+
	SLaz3	Human urine isolate (Braga)	+	+
*Proteus vulgaris*	ATCC 6380	Culture collection	-	+
	ATCC 6896	Culture collection	-	+
	ATCC 13315	Culture collection	+	+
	ATCC 29905	Culture collection	-	+
	SGSC 3359	Culture collection	+	+
	SGSC 5469	Culture collection	+	+
*Proteus hauseri*	ATCC 13315	Culture collection	-	+
*Proteus penneri*	ATCC 33519	Culture collection	+	+
*Citrobacter freundii*	SGSC 5345	Culture collection	-	-
*Citrobacter koseri*	CK18	Human pus isolate (Braga)	-	-
*Cronobacter sakazakii*	ATCC 29544	Culture collection	-	-
*Enterobacter aerogenes*	ATCC 13048	Culture collection	-	-
*Escherichia coli*	ATCC 11775	Culture collection	-	-
	CECT 434	Culture collection	-	-
	Ec7	Human urine isolate (Braga)	-	-
	Ec8	Human urine isolate (Braga)	-	-
	Ec9	Human urine isolate (Braga)	-	-
*Escherichia hermannii*	ATCC 33650	Culture collection	-	-
*Klebsiella pneumoniae*	ATCC 11296	Culture collection	-	-
*Morganella morganii*	SGSC 5703	Culture collection	-	-
	M12	Human urine isolate (Braga)	-	-
*Providencia rettgeri*	R1	Human sputum isolate (Braga)	-	-
*Providencia stuartii*	S7	Human urine isolate (Braga)	-	-
*Salmonella* Enteritidis	ATCC 13076	Culture collection	-	-
*Salmonella* Typhimurium	ATCC 43971	Culture collection	-	-

*^a^strain used on the enrichment procedure for phage isolation.*

### Phage Isolation, Production, and Titration

Bacteriophage isolation was performed using the enrichment procedure, essentially as described before (Melo et al., 2014b), using raw sewage (Braga). Briefly, 50 mL of centrifuged eﬄuent was mixed with the same volume of double-strength TSB and then inoculated with thirteen *P. mirabilis* strains (labeled with an “a” in **Table 1**). Fifty micro liter of each exponentially grown *P. mirabilis* culture were used. This solution was incubated for 18 h at 37°C, 120 rpm, centrifuged (10 min, 10,000 × *g*, 4°C) and the supernatant filtered through a 0.22 μm polyethersulfone membrane (GVS – Filter Technology). The presence of phages was checked by performing spot assays on bacterial lawns. Inhibition zones were purified to isolate all different phages on the respective bacterial host. Plaque picking was repeated until single-plaque morphology was observed and ten plaques of each isolated phage were measured and characterized.

Phage particles were produced using the plate lysis and elution method as described previously (Sambrook and Russell, 2001) with some modifications. Briefly, 10 μL phage suspension was spread on host bacterial lawns using a paper strip and incubated for 14–16 h at 37°C. After, 3 mL of SM Buffer [100 mM NaCl, 8 mM MgSO_4_, 50 mM Tris/HCl (pH 7.5), 0.002% (w/v) gelatin] were added to each plate and incubated for 8 h (120 rpm on a PSU-10i Orbital Shaker (BIOSAN), 4°C). Subsequently, the liquid and top-agar were collected, centrifuged (10 min, 10,000 × *g*, 4°C). The lysate was further concentrated with PEG 8000 and then purified with chloroform and stored at 4°C.

Phage titration was performed according to the double agar overlay technique (Kropinski et al., 2009). Briefly, 100 μl of diluted phage solution, 100 μl of host bacteria culture, and 3 mL of soft agar were poured onto a Petri plate containing a thin layer of TSA. After overnight incubation at 37°C, the plaque forming units (PFUs) were determined.

### Lytic Spectra of the Isolated Phages

The host range of the two isolated phages was determined by pipetting 10 μl of diluted phage solution (10^8^ PFU.mL^-1^) on lawns of indicated bacterial strains (**Table 1**). Plates were incubated 12 h at 37°C, and the presence and absence of inhibition zones indicating host sensitivity were reported.

### Electron Microscopy

The morphology of phage particles was observed by transmission electron microscopy, as previously described (Melo et al., 2014b). Briefly, phage particles were collected after centrifugation (1h, 25,000 × *g*, 4°C). The pellet was washed twice in tap water using the same centrifugation conditions. Phages were further deposited on copper grids with carbon-coated Formvar films, stained with 2% uranyl acetate (pH 4.0). Phages were observed using a Philips EM 300 electron microscope, and magnification was monitored with T4 phage tails (Ackermann, 2009).

### One-step Growth Curve

One-step growth curve studies were performed as described previously (Rahman et al., 2011), with some modifications. Briefly, 10 mL mid-exponential-phase culture, OD_620_ 0.5, was harvested by centrifugation (5 min, 7000 × *g*, 4°C) and resuspended in 5 mL fresh TSB medium in order to obtain an OD_620_ of 1.0. The same volume of phage solution was added in order to achieve a multiplicity of infection (MOI) of 0.005. After adsorption during 5 min (37°C, 120 rpm) the mixture was centrifuged as described above, and the pellet resuspended in 10 mL of fresh TSB. Samples were taken every 5 min over a period of 30 min and every 10 min until 1 h of infection.

### DNA Isolation, Genome Sequencing, and Annotation

Phage DNA was extracted as described before (Melo et al., 2014a). Purified phages were treated with 0.016% (v/v) L1 buffer [300 mM NaCl, 100 mM Tris/HCl (pH 7.5), 10 mM EDTA, 0.2 mg BSA mL^-1^, 20 mg RNase A mL^-1^ (Sigma), 6 mg DNase I mL^-1^ (Sigma)] for 2 h at 37°C. After a thermal inactivation of the enzymes for 30 min at 65°C, 50 μg proteinase K ml^-1^, 20 mM EDTA, and 1% SDS were added and proteins were digested for 18 h at 56°C. This was followed by phenol:chloroform:isoamyl alcohol solution (25:24:1, v/v) and chloroform extractions. DNA was precipitated with isopropanol and 3 M sodium acetate (pH 4.6), centrifuged (15 min, 7,600 × *g*, 4°C), and the pellet air-dried and further resuspended in nuclease-free water (Cleaver Scientific). Genome sequencing was performed on a 454 sequencing platform (Plate-forme d′ Analyses Génomiques at Laval University, Québec city, QC, Canada) to 50-fold coverage. Sequence data was assembled using SeqMan NGen4 software (DNASTAR, Madison, WI, USA). Phage genomes were autoannotated, using MyRAST (Aziz et al., 2008) and the presence of non-annotated CDSs, along with genes in which the initiation codon was miscalled, were checked manually using Geneious 6.1.6 (Biomatters). Potential frameshifts were checked with BLASTX (Altschul et al., 1997) and BLASTP was used to check for homologous proteins (Altschul et al., 1990), with an E value threshold of <1 × 10^-5^ and at least 80% query. Pfam (Finn et al., 2014) was used for protein motif search, with the same cutoff parameters as used with BLASTP. Protein parameters (molecular weight and isoelectric point) were determined using ExPASy Compute pI/Mw (Wilkins et al., 1999). The presence of transmembrane domains was checked using TMHMM (Kall and Sonnhammer, 2002) and Phobius (Kall et al., 2004), and membrane proteins were annotated when both tools were in concordance. The search of tRNA encoding genes was performed using ARAGORN (Laslett and Canback, 2004) and tRNAscan-SE (Schattner et al., 2005). Fragments 100 bp upstream of each predicted ORF were extracted and MEME (Bailey et al., 2009) was used to search for putative promoter regions that were further manually verified. For the same purposes, PHIRE was also used (Lavigne et al., 2004). ARNold (Naville et al., 2011) was used to predict rho-independent terminators and the energy was calculated using Mfold (Zuker, 2003). EMBOSS Stretcher (Rice et al., 2000) and CoreGenes (Zafar et al., 2002) were used for whole genome comparisons between *P. mirabilis* phages and their closest relatives. Phage 5460 was compared with Enterobacteria phages: K1-5 (NC_008152), UAB_Phi78 (NC_020414), and SP6 (AY370673). Phage 5461 comparisons were performed with *Yersinia* phage phiR1-RT (HE956709), and *Salmonella* phages STP4-a (KJ000058) and S16 (HQ331142). For phylogenetic analysis, homologous proteins were identified using BLASTP against the virus database at NCBI. A phylogenetic tree was constructed using “One Click” phylogeny.fr ^1^ (Dereeper et al., 2008). The data was exported in Newick tree format and opened in FigTree^2^.

### Evaluation of the Degree and Stability of Phage Coating

Silicone coupons were placed in 24-well plates and 1.7 mL of both phage solutions with different concentrations (10^6^, 10^7^, 10^8^, 10^9^, and 10^10^ PFU.mL^-1^) were added. Plates were incubated overnight at room temperature without agitation. Coupons were removed, washed with SM Buffer and placed in a new well with the same volume SM Buffer.

Different sonication conditions were optimized to assure the efficient removal of viral particles without compromising the particles viability. Accordingly, a loss of viability for both phages was observed with an increase of the sonication period (>5 s) and amplitude (loss of viability at 25% > loss of viability at 22%) (Supplementary Figure S1). While it seems that amplitude increase has a negative effect on phage viability, there were no statistical differences between the 22 and 25% of amplitude (*p* > 0.05). Statistical differences were only noticed between the different sonication times for phage 5461 (for both amplitudes) and for phage 5460 (only for 25% of amplitude) (*p* < 0.05). Consequently, coupons were further sonicated for 5 sec at 22% amplitude and PFU.mL^-1^ were determined. The sonication was optimized to assure the efficient removal of viral particles without compromising virus viability using two different amplitudes (22 and 25%, Sonics Vibra-Cell VC 505 – VC 750 sonicator) during different sonication times (5, 10, and 20 s).

To further evaluate the “natural” release of phages from the silicone surface overtime, phage-coated coupons, were exposed to AU supplemented with 0.3% glucose at 37°C. For the surface coating, the concentration of the initial suspension was adjusted to obtain a concentration of adhered phages ranging from 10^5^ to 10^6^ phages/cm^2^. At specific time points (2, 4, 6, 24, 48, 72, and 96 h) the number of released phages was determined by plaque assay.

### Screening of *P. mirabilis* Strains for Biofilm Formation Ability

The stability of the phages was assessed after incubating the phages on AU at 37°C for 168 h with phages samples titration every 24 h.

For biofilm formation assays, 15 *P. mirabilis* strains (**Table 1**) were grown in 10 mL of AU supplemented with 0.3% glucose, and incubated for 16–18 h (orbital shaker ES-20/60 (BIOSAN), 120 rpm, 37°C). Bacterial cultures were centrifuged (3 min, 5,000 × *g*) and pellets resuspended in fresh AU to an OD_620nm_ of 0.1 (∼1 × 10^8^ CFU.mL^-1^). Twenty μl of each culture were added to 180 μl of fresh AU in a 96-well plate (*n* = 6) and were incubated for 48 h, at the conditions described above, with media renewal after 24 h. Negative controls were performed with 200 μl of AU (*n* = 6).

Biofilm biomass was quantified as previously described (Pires et al., 2011), with some modifications. Briefly, after 48 h of incubation, all media was removed and the wells washed twice with phosphate buffered saline (PBS), pH 7.5. After PBS removal, 220 μl of methanol were added for 20 min, the methanol was removed, and the plates were air-dried. Then, 220 μL of 1% crystal violet (w/v, Merck) were added to each well, incubated for 10 min at room temperature, and washed with tap water. Finally, 220 μL of 33% acetic acid (v/v, Fisher) were added to each well to dissolve the stain and the absorbance measured at 570 nm, in an ELISA reader (Tecan). Three independent experiments were performed. Strains were classified regarding biofilm formation, as previously described (Stepanovic et al., 2000). A cut-off (OD_c_) was defined with three standard deviations above negative controls (AU). Four classes were defined: OD ≤ OD_c_ – non-biofilm forming strain; OD_c_ < OD ≤ 2 × OD_c_ – weak biofilm forming strain; 2 × OD_c_ < OD ≤ 4 × OD_c_ – moderate biofilm forming strain; 4 × OD_c_ < OD – strong biofilm forming strain.

### Efficacy of a Phage Cocktail under Dynamic Biofilm Formation Conditions

To mimic catheter conditions, biofilms were formed in a continuous model on Foley catheters (Silkemed Uro-cath 2Way, Overpharma). The flow was set to 0.5 mL.min^-1^ to mimic the actual average flow in a catheterized patient (Jones et al., 2005; Levering et al., 2014). With the exception of the catheter, all other tubing of the system was autoclaved (15 min, 121°C). Under sterile conditions and with the aid of a sterile scalpel the two ends of the Foley catheters were removed to allow its attachment to the other tubing of the system. A phage cocktail containing the same concentration of phages Pm5460 and Pm5461 was prepared. Three mL of the phage cocktail at 10^9^ PFU.mL^-1^ (to obtain a 10^6^ PFU.cm^-2^ coating) were added to each catheter, the catheter was sealed and phage binding to the catheter material allowed to occur overnight at room temperature under static conditions. On the following day, all non-bound phages were removed flowing the catheter with SM Buffer, the flow systems was mounted under sterile conditions, and then catheters were supplied with AU inoculated with 1 × 10^5^ CFU.mL^-1^ of both *P. mirabilis* SGSC 5446 and SGSC 5449 (previously grown for 16 h in 10 mL AU supplemented with 0.3% glucose, centrifuged (3 min, 5,000 × *g*) and suspended at the desired concentration). Phage-coated and non-coated catheters were supplied with a continuous flow of fresh urine with the bacterial suspension for 24 h, and after with AU for up to 168 h with no recirculation of the medium. At 48, 96, and 168 h, 2 cm of each catheter were removed: 1 cm for microscopy analysis, and 1 cm for CFU analysis. To determine the number of viable cells, samples were resuspended in 1.5 mL NaCl 0.9%, sonicated for 5 s at 22%, and plated in TSA plates. Four independent assays were performed. The sonication step was previously optimized to assure complete detachment with no loss of cellular viability (see Supplementary Figure S1). Briefly, to optimize the sonication conditions for the detachment of cells, 1 mL of a fresh inoculum of *P. mirabilis* set to an OD_620nm_ of 0.1 was added to each well of a 24-well plate and sonicated using two different amplitudes (22 and 25%, Sonics Vibra-Cell VC 505 – VC 750 sonicator) during different sonication times (5, 10, 15, 20, and 40 s). Viable cells at each time point and amplitude were determined. Three independent assays were performed. The best conditions were applied to biofilms samples, and efficient removal was confirmed by microscopy.

### Microscopy Observations of the Biofilms

Epifluorescence observations: To assess the ability of phages to control the biofilms formed on silicone catheters, catheter sections were stained with DAPI. Briefly, 0.5 cm sections of phage-coated or non-coated catheters taken at each time points were stained with 100 μl (100 μg.mL^-1^) of DAPI and placed in the dark for 10 min. The excess of dye was removed using absorbent paper, the catheter sections were placed on microscope slides and analyzed using an epifluorescence microscope (Olympus, Model BX51, Hamburg, Germany) equipped with a CCD camera (Olympus, Model DP72) with a filter-block for DAPI fluorescence (Ex 365–370; Barrier 400; Em LP 421).

Scanning electron microscopy (SEM) observations: SEM observations were performed on washed (PBS) catheter sections, which had been gradually dehydrated in absolute ethanol (Merck) solutions (15 min each in 10, 25, 40, 50, 70, 80, and 100% v/v). The catheter sections, kept in a desiccator until observed, were sputter coated with gold and observed with an S-360 scanning electron microscope (Leo, Cambridge, USA).

### Statistical Analysis

All graphs were generated using GraphPad Prism 5 software (GraphPad Software). Means and standard deviations (SD) were calculated. Statistical analysis was carried out by two-way repeated-measures analysis of variance (ANOVA) with Bonferroni *post hoc* tests. Both tests were performed used using GraphPad software. Differences between samples were considered statistically different for *p*-values lower than 0.05.

### Nucleotide Sequence Accession Numbers

The complete genome sequences of the two *P. mirabilis* phage isolates were submitted to the GenBank under the accession numbers KP890822 (vB_PmiP_5460) and KP890823 (vB_PmiM_5461).

## Results

### Isolation and Morphology of Proteus mirabilis Phages

**FIGURE 1:**
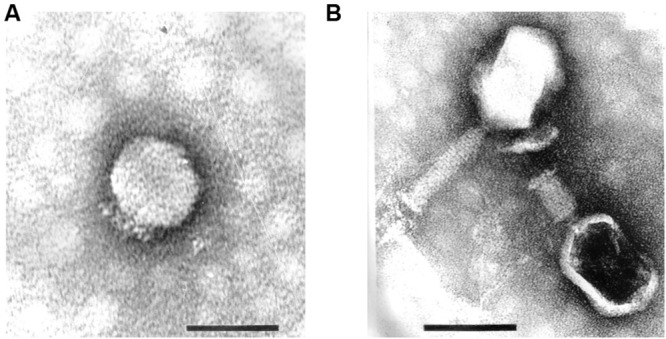
**Transmission electron micrographs of *Proteus mirabilis* phages: **(A)** Pm5460; **(B)** Pm5461.** Scale bars represent 100 nm.

These strains were used for phage enrichment (**Table 1**). In the initial screening, two different plaque morphotypes were detected. These plaques were purified and two different phages were isolated – vB_PmiP_5460 (5460) and vB_PmiM_5461 (5461), using *P. mirabilis* SGSC 5460 and SGSC 5461 as hosts, respectively. According to the morphological evaluation, phage 5460 belongs to the *Podoviridae* family of phages having a capsid 65 nm in diameter, a short (13 nm) tail terminating in tail fibers (**Figure 1A**). Phage 5461 has isometric head 87 nm in diameter, and a contractile tails (110 nm long and 17 nm wide) and belongs to the *Myoviridae* family (**Figure 1B**). Phage 5460 forms clear plaques 1.5 mm in diameter while phage 5461 forms smaller (1 mm) and less clear plaques.

### Host Range Screening

To investigate the host specificity of both phages, a total of 43 strains of *Enterobacteriaceae* listed in **Table 1** were used. Phage 5460 lysed 12 out of 18 *P. mirabilis* strains (67%), three out of six *P. vulgaris* strains and one tested *P. penneri* strain; while phage 5461 killed all (100%) of the *Proteus* spp. tested. Notwithstanding the fact that only 43 strains were tested, both phages seem to have a *Proteus*-genus-specific profile, having no activity against other *Enterobacteriaceae* strains. It is likely that phage 5461 recognizes a conserved outer membrane protein (OMP) or polysaccharide such as the inner core region of lipopolysaccharide (LPS) which is known to be conserved among at least *P. mirabilis* strains (Vinogradov and Perry, 2000; Kaca et al., 2011).

### One-step Growth Curve

The replication of phages 5460 and 5461 on their respective hosts (Supplementary Figure S2) revealed a latent and rise periods of phage 5460 of approximately 10 and 15 min, and an average burst size of 46 PFU per infected cell. On the other hand, phage 5461 showed a latency period of 25 min, a rise period of 10 min and a burst size of 11 PFU per infected cell. Within the 60 min of duration of the experiment, a second cycle of replication was evident for both phages.

### Genome Analysis

Genome analysis revealed that both phages are virulent, not encoding any genes associated with lysogeny. Furthermore, no known virulence-associated or toxic proteins were detected *in silico*, revealing that both phages are potentially safe for therapeutic purposes.

Phage 5460 has a linear double-stranded DNA with 44,573 bp with a G+C content of 39.6% (**Table 2**). This phage encodes 53 putative CDSs, tightly packed occupying 93% of its genome. Of the CDSs, 20 have an assigned function and 11 are unique (Supplementary Table S1A). No tRNA genes were detected. The majority (90.6%) of the CDSs possess methionine as start codon, while only 5% a GTG start codon. BLASTN searches revealed that 5460 is homologous to the *Autographivirinae* phages: K1–5 (*Escherichia coli*), UAB_Phi78 (*Salmonella* Typhimurium) and SP6 (*Salmonella* Typhimurium). The comparison of the nucleotides of 5460 and these three phages with EMBOSS stretcher showed that 5460 has identities around 60% with these phages, being most similar (63.4%) to phage SP6 while CoreGenes (40) analysis showed that these phages share 33 homologs, which represent 62.3% of the 5460 proteins. Furthermore, the phylogenetic tree of the DNA polymerase containing homologs to 5460, demonstrated that this phage shares a branch with *Proteus* phages PM_85 and PM_93, being very close to the Enterobacteria phage SP6 (Supplementary Figure S3A). Consequently, this phage can be assigned to the *Sp6virus* genus.

**Table 2:** *Proteus mirabilis* phages genome properties.

Phage	Genome size (Kb)	G+C content (mol%)	Putative CDSs	Promoters/terminators	tRNAs	Closest homolog (% identity)
Pm5460	44,573	39.6	56	10/5	0	Enterobacteria phage SP6 (63.4)
Pm5461	161,989	31.1	256	34/15	8	*Yersinia* phage phiR1-RT (53.1)

Phage 5461 has a linear double-stranded DNA with 161,989 bp with a GC content of 31.1%, codifying 256 putative CDSs that occupy 95.2% of the genome (**Table 2**). From these CDSs, 123 have assigned function, while 90 are unique having no significant homologies to any proteins on public databases (Supplementary Table S1B). Ninety-five percent of the CDSs have an ATG as a start codon, while TTG (3%) and GTG (2%) are also used by this phage. This phage encodes eight different tRNA genes (tRNA-Glu, tRNA-Ser, tRNA-Asp, tRNA-Gly, tRNA-Pro, tRNA-Met, tRNA-Tyr, tRNA-Arg). BLASTN searches showed that the *Yersinia* phage phiR1-RT and *Salmonella* phages STP4-a and S16 are the closest relatives and therefore it can be assumed that this phage is part of the *Tevenvirinae* subfamily. EMBOSS stretcher alignment showed that 5461 and *Yersinia* phage phiR1-RT have an identity of 53.1%, while 5461 shares 52.2% of identity with *Salmonella* phages STP4-a and S16. CoreGenes (40) results showed that 5461 shares 133 homologous proteins with these three phages, which represent 45.3% of total proteins of 5461. The phylogenetic tree comprising the homologs of 5461 terminase large subunit shows that 5461 along with *Serratia* phage PS2 and *Citrobacter* phage Merlin are between the two ICTV approved taxa – *Js98virus* and *Cc31virus* (Supplementary Figure S3B).

### Screening of *P. mirabilis* Strains for Biofilm Formation Ability

Phage stability in AU was assessed and both phages were shown to keep their titer after incubation in AU for 168 h (data not shown).

**FIGURE 2:**
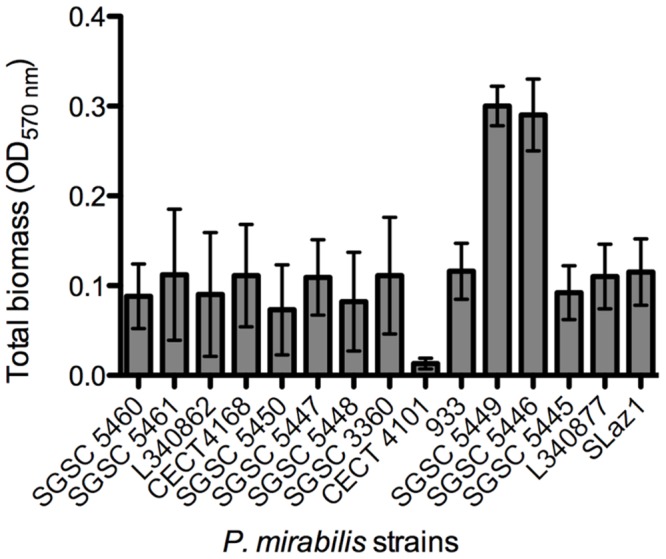
**Screening for biofilm formation of fifteen *P. mirabilis* isolates on 96-well microtiter plates using crystal violet staining.** Data points represent an average of three independent experiments performed in triplicate. Error bars indicate standard deviation.

Then, an initial screening was performed to select strong biofilm-forming *P. mirabilis* strains according to the classification reported by Stepanovic et al. (2000). In total, biofilms of 15 different *P. mirabilis* strains were formed in AU for 48 h and the total biofilm biomass quantified (**Figure 2**). The vast majority of the strains were classified as moderate biofilm forming, five were defined as weak biofilm forming strains, *P. mirabilis* CECT 4101 was the only strain classified as a non-biofilm forming strain, and *P. mirabilis* strains SGSC 5449 and SGSC 5446 were the only strong biofilm-forming strains.

### Evaluation of the Amount of Phages Coating the Silicone Surface

**FIGURE 3:**
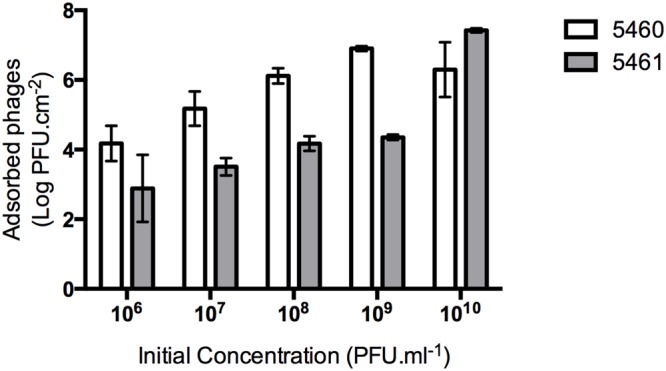
**Number of phages coating silicone surfaces per surface area.** Data points represent an average of three independent experiments performed in triplicate and error bars indicate standard deviation. Statistical differences (*p* < 0.05) between the phage initial concentration and adsorbed phages were determined by two-way repeated-measures analysis of variance (ANOVA) with Bonferroni *post hoc* test.

An efficient phage coating of the surfaces is critical to assure the success of an anti-biofilm strategy. Physical adsorption has been used to immobilize bacteriophages (Hosseinidoust et al., 2011), however, it cannot be assured a correct orientation of the phage (as tails must be available to interact with bacteria; Cademartiri et al., 2010). To better evaluate the effectiveness of phage physical adsorption, different phage concentrations were placed in contact with silicone surfaces (**Figure 3**). The amount of phages retained at the silicone surfaces increased proportionally to the initial concentration. The amount of 5460 phage particles adsorbed to the silicone surfaces was in average 1 log higher than the amount of 5461 phages (**Figure 3**).

### Evaluation of Phage Release from Silicone Surfaces

**FIGURE 4:**
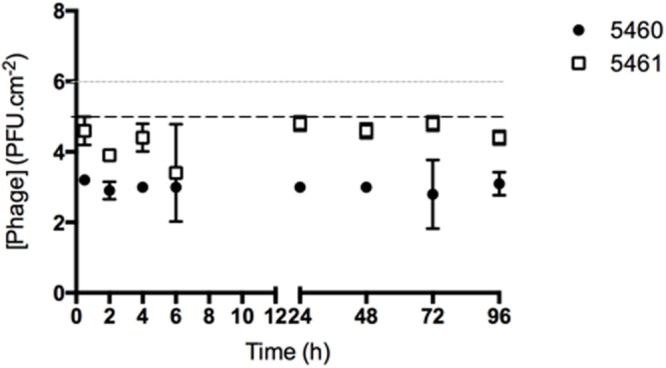
**Number of phages released from silicone surfaces per surface area.** Data points represent an average of three independent experiments performed in triplicate and error bars indicate standard deviation. The approximate concentration of adhered 5460 phage is presented in the graphic as a black dot intermittent gray line, while approximate concentration of adhered 5461 phage is presented as an intermittent black line. Statistical differences (*p* < 0.05) between the approximate initial phage titer with released phage were determined by two-way repeated-measures analysis of variance (ANOVA) with Bonferroni *post hoc* test.

The number of phages released from silicone surfaces was determined over time (**Figure 4**). The amount of phages 5460 released from the surfaces was in the range of 10^3^ PFU.mL^-1^ (around 1% of the adhered phages). Phage 5461 was almost completely released from the surface after 2 h in contact with AU.

### Dynamic Model for Biofilm Formation

**FIGURE 5:**
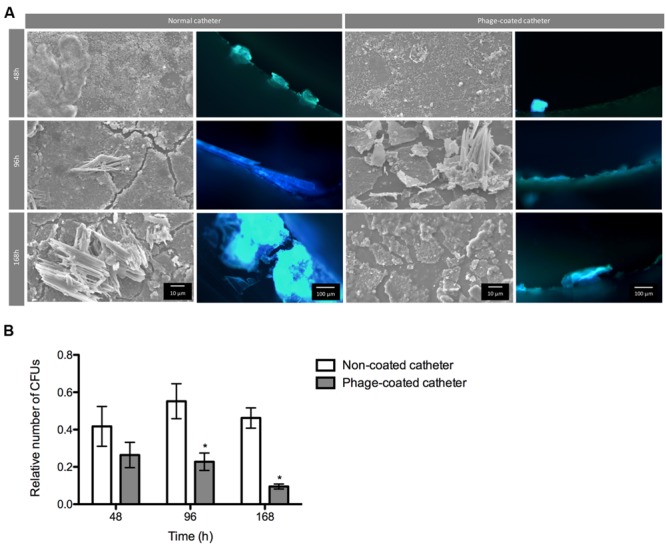
**Phage cocktail effect on *P. mirabilis* biofilms under dynamic conditions.** Representative SEM and epifluorescence images **(A)** and relative number of CFU **(B)** of *P. mirabilis* biofilms formed in normal and phage-coated Foley catheter show a decrease on the biofilm population, as well as on the overall biomass. SEM images are not representative of the overall biofilm as they are focused on spaces where biofilm is present. Relative CFU values were normalized using the higher concentration of biofilm cells obtained in each independent experiment. Magnification of 100x and 1000x, were used for epifluorescence and SEM images, respectively. Data points represent an average of four independent experiments and error bars indicate standard deviation. Statistical differences (*p* < 0.05) between control biofilms and phage-cocktail- -treated biofilms (^∗^) were determined by two-way repeated-measures analysis of variance (ANOVA) with Bonferroni *post hoc* test.

The conditions found in real urinary catheters were mimicked using a continuous biofilm model system using Foley catheters. The viable cell counts and microscopy imaging of phage cocktail-coated catheters were compared with the control (non-phage coated) catheters (**Figure 5**). The higher concentration of biofilm cells obtained in each independent experiment was used to normalize the data.

During the assays, bacterial concentrations ranged between log 6 and log 8. According to the results, the phage-coating led to the reduction of the biofilm population and that difference was even more evident with the increase of biofilm age. Despite the non-significant differences of viable cells after 48 h between the phage-coated or non-coated catheters sections, a clear tendency of the phage cocktail to reduce *P. mirabilis* biofilms was already observable (**Figure 5**) leading to significant reductions (*p* < 0.05) at 96 and 168 h. This was also confirmed by epifluorescence and SEM (**Figure 5**) microscopy where more cells were observed in control catheter sections than in phage-coated catheters (**Figure 5**).

## Discussion

Urinary tract infections are involved in 40% of all nosocomial infections (Saint et al., 2008). Although Gram-positive bacteria, such as *Staphylococcus epidermidis* and *Enterococcus faecalis* can cause these infections, Gram-negative *Enterobacteriaceae*, are the most commonly implicated in CAUTI development (Siddiq and Darouiche, 2012). *P. mirabilis* forms crystalline biofilms within the urinary tract and is responsible for up to 30% of all urinary tract stones (struvite) (Stickler and Morgan, 2006). Furthermore, these crystalline structures recurrently block the flow through catheters (Stickler and Feneley, 2010). As frequently observed with different bacterial species, *P. mirabilis* strains are important reservoirs of antibiotic resistant determinants (Harada et al., 2014) and, because of this, resistant phenotypes have emerged in last years (Harada et al., 2014; Wang et al., 2014). Furthermore, as in other species, *P. mirabilis* biofilms were shown to be more tolerant to several antibiotics, than their planktonic counterparts (Moryl et al., 2013).

On the last decades the use of phages as alternatives or complements to antibiotic therapy has extensively been evaluated (Viertel et al., 2014) and has even been listed by the US National Institute of Allergy and Infectious Diseases as one important approach to combat antibiotic resistance (Reardon, 2014). The current relevance of phage therapy associated with the lack of *P. mirabilis* phages described so far, were the underlying reasons for this study.

Two *P. mirabilis* phages, isolated from raw eﬄuents of wastewater treatment plants, were characterized. TEM analysis demonstrated that both belong to the *Caudovirales* order and are, respectively, members of the *Podoviridae* (phage 5460) and the *Myoviridae* (phage 5461) families. Their spectrum of activity against a collection of *Proteus* spp. was 16 out of 26 strains for phage 5460 and 26 out of 26 strains for 5461. Both phages were unable of killing other Enterobacteriaceae strains tested. Despite both phages have shown specificity to *Proteus* genera, it should be noted that only a limited number of strains of other species was used. As expected, these two phages had clear differences in the replication parameters, which resulted in higher burst size for the *Podoviridae* phage.

Based upon the genome sequence, phage 5460 belongs to the *Autographivirinae* subfamily, specifically to the *Sp6virus* genus. The GC content of phage 5460 is 39.6%, a value similar to *P. mirabilis* HI4320 strain (GC 38.9%) and to *Proteus* phage PM16 (41.4%G+C) and 5460 shares protein homology with enterobacterial *Podoviridae* phages K1-5 (63.5%), SP6 (63.5%), and UAB_Phi78 (56.9%).

Despite the morphological similarities with *Myoviridae* of the T4-like phages, phage 5461 has different characteristics from those deposited in GenBank. For instance, the GC content of 5461 is 31.1%, which is clearly lower than the content observed in other T4-like viruses and in its host species. This low GC content may cause codon usage problems during phage infection (Rocha and Danchin, 2002). The presence of eight tRNA genes on 5461 genome might attenuate these differences, as the presence of tRNAs in phage genomes is correlated with the differences in the codon usage between the phage and the host, corresponding to codons that are expected to be poorly translated by the host machinery (Bailly-Bechet et al., 2007). Phage 5461 shares homologous proteins with the myoviruses *Yersinia* phage phiR1-RT (44.3%) and *Salmonella* phages STP4-a (45.1%) and S16 (43.3%). Furthermore, the phylogenetic analysis have shown that this phage cannot be assigned to a known genus.

The two phages characterized were studied for their potential preventive activity toward biofilms. For this, silicone surfaces were coated with phages using standard adsorption procedure. The fact that phage 5460 adsorbed in greater amounts than phage 5461 can be due to the different morphological characteristics and composition of the phages. Hosseinidoust et al. (2011), described that the morphology of the phages has a great influence on phage adsorption due to their orientation on the surface. Podoviruses are shorter that myoviruses, therefore the ratio between the surface area and the phage length is greater for podophages, consequently it is expected that a greater quantity of 5460 are adsorbed to silicone surfaces.

In order to obtain a stable coating it is important that phages remained attached to the surface during flow (Lehman and Donlan, 2015), therefore phage release from surfaces was also evaluated by titer determination. The results pointed out to a greater adhesion strength between phage 5460 and the silicone surface compared to phage 5461. It has been previously shown that phages from different families have different surface-coating properties (Hosseinidoust et al., 2011). However, the reason why the coating stability differs between phages is not apparent as the phages major capsid proteins – the most abundant component of the phage particle - present similar isoelectric points (phage 5460: 5.15; phage 5461: 5.10) and hydrophibicities (phage 5460: -0.23; phage 5461: -0.33), which would suggest the same ability to interact with the surface. Although the majority of the adhered phages are released in short periods, the host can colonize the catheter surface forming biofilms. At this stage, not only the microbial cells adhered to the catheter surface, but also the biofilm by itself are phage reservoirs (Doolittle et al., 1996). Therefore, phages within the biofilm will be available to infect neighbor cells delaying biofilm formation and development.

After performing a rapid screening for biofilm formation in different *P. mirabilis* strains, two good biofilm-forming strains were selected for further tests in dynamic models. The two good biofilm-forming strains have shown similar susceptibly to both phages and thus have provided a suitable combination for the dynamic studies.

Due to their distinct lytic spectra and surface binding characteristics, a cocktail of both phages was used to prevent biofilm formation under dynamic conditions. Since urinary catheters can stay inserted in the patients’ bladder for long periods, the efficacy of phage-coated catheters was prolonged up to 7 days. In fact, the phage cocktail developed herein reduced significantly the total number of cultivable cells after 96 and 168 h.

An important feature of *P. mirabilis* biofilms is its great ability to form crystals, which associated with bacterial cells attached to surfaces can lead to clogging of the catheter (Coker et al., 2000; Stickler et al., 2006). During our experiments this ability was evident as crystalline biofilms were seen microscopically and even by simple observation of the tubing at naked eye. Indeed, the difference in biofilm biomass in phage-coated or non-coated catheters were also apparent macroscopically.

Other authors have evaluated the use of a phage cocktail in the prevention and eradication of *E. coli* and *P. mirabilis* biofilms (Carson et al., 2010) and observed significant reductions (3 to 4 log) in the *E. coli* population, however, only a 1 log reduction was observed for the *P. mirabilis* population. These authors suggested that this lower efficacy might be related with phage-dependent factors, such as the production of depolymerases and the ability to penetrate the EPS matrix. In the case of the phage cocktail developed herein, this can also be one of the reasons for the low reductions (1 log) observed for the *P. mirabilis* biofilm. More recently, Lehman and Donlan (2015) used a phage cocktail on the pre-treatment of hydrogen-coated silicone catheters to prevent the adhesion of *Pseudomonas aeruginosa* and *P. mirabilis*. After treatment, the authors detected a higher decrease on CFU counts of *P. aeruginosa* than of *P. mirabilis*. Nonetheless, reduction in the *P. mirabilis* population was still pronounced with a log reduction of approximately 2, for both single and mixed biofilms.

Other reasons that might be hampering phage efficacies might be physico-chemical factors such as pH and ions (Jonczyk et al., 2011) and also the fact that the adsorption of phages to their target cells may be inhibited by the crystals formed in AU during biofilm formation. Furthermore, the biofilm matrix itself can block the specific phage receptors, preventing phage infection from occurring (Samson et al., 2013). Finally, the physiological state of the cells could also be influencing phage efficacy, since log-phase cells are faster and more efficiently lysed than cells with lower metabolic rates, such as the ones that compose biofilms (Hadas et al., 1997).

The specificity of the phages, their genome features, which do not encode genes associated with lysogeny, together with the moderate reductions obtained using *in vitro* simulated conditions, support the use of both phages vB_PmiP_Pm5460 and vB_PmiM_Pm5461 for preventing *P. mirabilis* biofilm formation on silicone catheters.

## Author Contributions

NC, CA, JA, and SS conceived the study. AK, CA, JA, and SS analyzed data. LM and PV performed experiments. LM wrote the paper. All authors read and approved the final manuscript.

## Conflict of Interest Statement

The authors declare that the research was conducted in the absence of any commercial or financial relationships that could be construed as a potential conflict of interest.

## Footnotes

1 http://phylogeny.lirmm.fr/phylo_cgi/simple_phylogeny.cgi

2 http://tree.bio.ed.ac.uk/software/figtree/
