# Supplementary material for: Development of a Phage Cocktail to Control Proteus mirabilis Catheter-associated Urinary Tract Infections
Source: Front Microbiol. 2016 Jun 28;7:1024. doi: 10.3389/fmicb.2016.01024 (PMC4923195; doi:10.3389/fmicb.2016.01024)
Supplement: Supplementary file 1 [file Table_1.PDF]

Table S1a. Features of the predicted CDSs of bacteriophage 5460

DNA				Protein							
CDS	Coordinates	Strand	Length	Mass	pI	aa residues	Function	Homolog	E-value	Motif	E-value
1	794..1072	+	279	10.65	10.09	92	hypothetical protein				
2	1183..1392	+	210	8.40	4.28	69	hypothetical protein				
3	1401..1517	+	117	4.17	4.68	38	hypothetical membrane protein	1 TMD			
4	1668..2168	+	501	20.00	4.41	166	hypothetical protein				
5	2189..2536	+	348	13.14	5.06	115	hypothetical protein	hypothetical protein vB_AbaM_Acibel004_78 [Acinetobacter phage vB_AbaM_Acibel004]	2,00E-14		
6	2706..2930	+	225	7.70	8.08	74	hypothetical protein				
7	3001..3846	+	846	31.95	5.18	281	hypothetical protein	gp7 [Enterobacteria phage SP6]	7,00E-100		
8	3920..6547	+	2628	99.63	6.31	875	RNA polymerase	RNA polymerase [Enterobacteria phage T3]	2,00E-114	polymerase N-terminal;	100
9	6849..6977	+	129	5.18	10.49	42	hypothetical protein	[Enterobacteria phage K1E]	2,00E-09		5,00E-15
10	6979..8967	+	1989	74.78	5.92	662	DNA primase/helicase	DNA primase [Enterobacteria phage SP6]	0.0	DnaB_C[PF03796]; DnaB helicase	3.1e-12
11	8967..9143	+	177	6.48	5.88	58	hypothetical membrane protein	1 TMD			
12	9215..9823	+	609	23.32	9.17	202	hypothetical protein	hypothetical protein PK1Ep15 [Enterobacteria phage K1E]	2,00E-36		
13	9823..10065	+	243	9.41	5.21	80	hypothetical protein	hypothetical protein ACG-C91_0016 [Enterobacteria phage vB_EcoP_ACG-C91]	5,00E-06		
14	10055..10216	+	162	6.09	9.52	53	hypothetical protein	hypothetical protein PK1Ep17 [Enterobacteria phage K1E]	9,00E-18		
15	10292..10444	+	153	5.62	4.11	50	hypothetical protein				
16	10431..12980	+	2550	97.20	6.01	849	DNA polymerase	putative DNA polymerase [Enterobacteria phage K1E]	0.0	DNA_pol_A[PF00476]; DNA polymerase	4.6e-20
17	13019..13564	+	546	20.98	5.34	181	hypothetical protein	hypothetical protein PP1_018 [Pectobacterium phage PP1]	1,00E-49	PolyA_pol[PF01743]; Poly A polymerase head domain	1.6e-06
18	13567..13797	+	231	8.69	9.41	76	hypothetical protein				
19	13816..13971	+	156	5.54	6.07	51	hypothetical protein				
20	13984..14790	+	807	29.39	4.79	268	hypothetical protein	gp18 [Enterobacteria phage SP6]	2,00E-160		
21	14794..15018	+	225	8.42	6.16	74	hypothetical protein	hypothetical protein phD2B_0018 [Lelliottia phage phD2B]	5,00E-15		
22	15121..15492	+	372	13.19	4.26	123	hypothetical protein	hypothetical protein phD2B_0019 [Lelliottia phage phD2B]	2,00E-49		

22A	15559..15804	+	246	9.67	5.58	81	hypothetical protein	hypothetical protein Phi78p27 [Enterobacteria phage UAB_Phi78]	2,00E-06		
23	15750..16400	+	651	24.28	5.07	216	exonuclease	exonuclease [Enterobacteria phage UAB_Phi78]	3,00E-119		
24	16782..17192	+	411	15.47	9.86	136	endonuclease	gp22 [Enterobacteria phage SP6]	5,00E-76	Endonuclease_7[PF02945]; Recombination endonuclease VII	8.6e-14
25	17185..18192	+	1008	37.91	7.04	335	hypothetical protein	hypothetical protein PK1Ep33 [Enterobacteria phage K1E]	0.0		
26	18259..18828	+	570	21.40	5.14	189	hypothetical protein	hypothetical protein P100_00280 [Erwinia phage phiEa100]	4,00E-48		
27	18825..19766	+	942	35.48	6.10	313	DNA ligase	DNA ligase [Enterobacteria phage UAB_Phi78]	6,00E-163	DNA_ligase_A_M[PF01068]; ATP dependent DNA ligase domain; DNA_ligase_OB_2[PF14743]; DNA ligase OB-like domain	3.5e-07; 4.6e-13
28	19759..20004	+	246	9.23	4.56	81	hypothetical protein				
29	19997..20185	+	189	6.73	3.99	62	hypothetical protein	hypothetical protein ACG-C91_0029 [Enterobacteria phage vB_EcoP_ACG-C91]	2,00E-20		
30	20154..20327	+	174	6.59	7.78	57	hypothetical protein	26 [Enterobacteria phage SP6]	2,00E-18		
31	20327..20821	+	495	18.76	5.81	164	acetyltransferase	hypothetical acetyltransferase [Enterobacteria phage K1E]	2,00E-67	Acetyltransf_1[PF00583]; Acetyltransferase	1.2e-06
32	20796..21002	+	207	7.26	9.77	68	hypothetical protein	28 [Enterobacteria phage K1-5]	2,00E-24		
33	21002..22552	+	1551	57.78	5.48	516	head-tail connector	head portal protein [Enterobacteria phage SP6]	0.0	Head-tail_con[PF12236]; Bacteriophage head to tail connecting protein	2.1e-120
34	22552..23433	+	882	32.25	4.20	293	scaffolding protein	putative scaffolding protein [Enterobacteria phage vB_EcoP_ACG-C91]	3,00E-113		
35	23507..24718	+	1212	43.79	5.15	403	major capsid protein	putative major capsid protein [Enterobacteria phage K1E]	0.0		
36	24774..25502	+	729	27.47	5.94	242	tail tubular A protein	tail protein [Enterobacteria phage SP6]	6,00E-133		
37	25504..27909	+	2406	90.65	5.40	801	tail tubular B protein	tail protein [Enterobacteria phage SP6]	0.0		
38	27909..28586	+	678	23.52	7.97	225	internal virion protein	internal virion protein [Enterobacteria phage UAB_Phi78]	7,00E-63		
39	28595..31546	+	2952	108.97	5.52	983	hypothetical protein	gp36 [Enterobacteria phage SP6]; lysozyme domain-containing protein [Pectobacterium phage PP1]	0.0		
40	31614..35435	+	3822	139.95	6.52	1273	internal virion protein	internal virion protein [Enterobacteria phage UAB_Phi78]	0.0		
41	35435..36394	+	960	36.27	5.42	319	tail protein/adaptor protein	putative tail fiber protein [Enterobacteria phage vB_EcoP_ACG-C91]	2,00E-139	Phage_T7_tail[PF03906]; Phage T7 tail fibre protein	5.5e-24
42	36404..36592	+	189	7.23	6.70	62	holin	putative holin [Enterobacteria phage K1E]; 1 TMD	3,00E-27	Phage_holin_6[PF10746]; Phage holin family 6	1.4e-05

43	36579..36884	+	306	10.84	5.60	101	terminase, small subunit	putative small terminase subunit [Enterobacteria phage K1E]	8,00E-49		
44	36884..38782	+	1899	72.15	6.40	632	terminase, large subunit	large terminase subunit [Enterobacteria phage SP6]	0.0		
45	38949..39230	+	282	9.93	5.66	93	hypothetical protein	hypothetical protein PK1Ep55 [Enterobacteria phage K1E]	2,00E-20		
46	39242..39526	+	285	10.01	8.01	94	hypothetical protein	42 [Enterobacteria phage K1-5]	6,00E-41		
47	39535..39888	+	354	13.14	9.48	117	peptidase	putative M15 family peptidase [Lelliottia phage phD2B]	9,00E-62	Peptidase_M15_3[PF08291]; Peptidase M15	4,00E-33
48	39911..40138	+	228	7.68	8.76	75	hypothetical membrane protein	44 [Enterobacteria phage K1-5]; 1 TMD	1,00E-09		
49	40131..40328	+	198	7.28	9.10	65	hypothetical protein	hypothetical protein ACG-C91_0051 [Enterobacteria phage vB_EcoP_ACG-C91]	5,00E-06		
50	40303..40419	+	117	4.43	6.06	38	hypothetical protein	hypothetical protein PK1Ep59 [Enterobacteria phage K1E]	1,00E-12		
51	40556..42805	+	2250	82.33	6.48	749	tailspike protein	putative hemagglutinin protein [Listonella phage phiHSIC]	9,00E-47		
52	42890..44461	+	1572	56.91	6.36	523	hypothetical protein				

Table S1b. Features of the predicted ORFs of bacteriophage 5461

	DNA		Protein								
**CDS**	**Coordinates**	**Strand**	**Length**	**Mass**	**pI**	**aa residues**	**Function**	**Homolog**	**E-value**	**Motif**	**E-value**
1	1..2046	-	2046	78.7	5.66	681	RIIA	RIIA [Salmonella phage S16]	3,00E-95		
2	2073..2264	-	192	7.37	4.86	63	hypothetical protein				
3	2274..2489	-	216	8.27	6.07	71	hypothetical protein				
4	2486..2866	-	381	15.08	7.74	126	topoisomerase II, large subunit, C-terminal region	gp60 topoisomerase II, large subunit, C-terminal region [Enterobacteria phage T4]	8,00E-45		
5	2882..3022	-	141	5.23	5.97	46	topoisomerase II, large subunit, C-terminal region	DNA topoisomerase II large subunit C-terminal region [Salmonella phage S16]	4,00E-11		
6	3000..3809	-	810	31.32	9.91	269	homing endonuclease	homing endonuclease [Enterobacteria phage T4T]	2,00E-96	HNH[PF01844]; HNH endonuclease	9.2e-05
7	3796..5340	-	1545	57.63	7.09	514	topoisomerase II, large subunit, N-terminal region	gp39 topoisomerase II, large subunit, N-terminal region [Enterobacteria phage T4]	0.0	HATPase_c[PF02518]; Histidine kinase-, DNA gyrase B-, and HSP90-like ATPase; DNA_gyraseB[PF00204]; DNA gyrase B	3e-11; 5.7e-26
8	5356..5694	-	339	13.71	5.60	112	hypothetical protein				
9	5728..6162	-	435	16.74	4.54	144	hypothetical protein				
10	6164..6586	-	423	16.52	6.30	140	hypothetical protein				

11	6589..7026	-	438	16.96	6.09	145	hypothetical protein				
12	7023..7961	-	939	36.15	4.65	312	hypothetical protein	hypothetical protein PHG31p11 [Aeromonas phage 31]	6,00E-13		
13	7966..8181	-	216	8.10	4.77	71	cef protein	cef [Aeromonas phage 31]	3,00E-08		
14	8275..8952	-	678	26.08	5.14	225	exonuclease A	DexA exonuclease A [Enterobacteria phage T4]	2,00E-88		
15	8949..10271	-	1323	50.97	6.30	440	DNA helicase	DNA helicase, phage-associated [Yersinia phage phiR1-RT]	2,00E-156	AAA_30[PF13604]; AAA proteins	0,00058
16	10268..10561	-	294	11.91	9.54	97	hypothetical protein	hypothetical protein Ac42p025 [Acinetobacter phage Ac42]	1,00E-05		
17	10518..11177	-	660	25.80	9.85	219	anti-sigma factor	putative anti-sigma factor [Serratia phage PS2]	2,00E-19		
18	11234..11428	-	195	7.39	9.35	64	hypothetical protein				
19	11428..11952	-	525	20.01	4.78	174	dCTP pyrophosphatase	dCTP pyrophosphatase [Enterobacter phage PG7]	1,00E-82	dUTPase_2[PF08761]; DUTP diphosphatase; dUTPase_2[PF08761]; DUTP diphosphatase	8.8e-20; 1.1e-06
20	11962..12540	-	579	21.99	5.01	192	hypothetical protein			MIB_HERC2[PF06701]; Mib_herc2	1.2e-05
21	12592..12891	-	300	11.36	7.91	99	hypothetical protein				
22	12893..13342	-	450	16.79	4.82	149	hypothetical protein	hypothetical protein Aeh1p322 [Aeromonas phage Aeh1]	4,00E-16		
23	13329..14117	-	789	30.34	6.62	262	hypothetical protein	hypothetical protein BN80_089 [Yersinia phage phiR1-RT]	7,00E-81		
24	14179..14364	-	186	6.94	4.23	61	hypothetical protein	hypothetical protein CC2_410 [Aeromonas phage CC2]	4,00E-05		
25	14351..14665	-	315	12.13	5.63	104	hypothetical protein				
26	14668..15687	-	1020	39.42	8.20	339	DNA primase subunit	DNA primase [Serratia phage PS2]	9,00E-124	Toprim_N[PF08275]; DNA primase catalytic core, N-terminal domain	8.4e-05
27	15738..16109	-	372	13.83	7.86	123	hypothetical protein	hypothetical protein PG7_030 [Enterobacter phage PG7]	3,00E-22		
28	16106..17527	-	1422	53.11	5.92	473	replication and recombination DNA helicase	DNA primase/helicase [Salmonella phage STML-198]	0.0	DnaB_C[PF03796]; DnaB helicase	3.9e-11
29	17527..17844	-	318	12.25	4.85	105	head vertex assembly chaperone	head vertex assembly chaperone [Salmonella phage S16]	1,00E-12	Phage_head_chap[PF11113]; Head assembly gene product	5,00E-19
30	17831..19210	-	1380	53.32	9.28	459	Hef-like homing endonuclease	putative Hef-like homing endonuclease [Acinetobacter phage Acj9]	2,00E-110		
31	19207..19830	-	624	23.77	4.87	207	RecA-like recombination protein	UvsX RecA-like recombination protein [Enterobacteria phage T4]	1,00E-79		
32	19845..20567	-	723	27.80	9.63	240	homing endonuclease	unnamed protein product [Enterobacteria phage T4]	6,00E-16	NUMOD3[PF07460]; NUMOD3 motif (2 copies)	6.2e-07

33	20611..21534	-	924	33.86	5.57	307	RecA-like recombination protein	RecA-like recombination protein [Enterobacteria phage CC31]	2,00E-123	RecA[PF00154]; recA bacterial DNA recombination protein	7,00E-08
34	21584..22498	-	915	35.77	5.18	304	hypothetical protein	hypothetical protein EpJS98_gp038 [Enterobacteria phage JS98]	6,00E-103		
35	22507..23196	-	690	26.63	5.12	229	thymidylate synthase	putative thymidylate synthase [Escherichia phage vB_EcoM_PhAPEC2]	1,00E-125	Thymidylat_synt[PF00303]; Thymidylate synthase	7.2e-24
36	23193..23579	-	387	14.90	9.36	128	hypothetical protein			CM_2[PF01817]; Chorismate mutase	4.6e-10
37	23636..23845	-	210	7.90	10.06	69	hypothetical protein				
38	23817..24983	-	1167	45.26	6.34	388	peptidase	putative peptidase [Escherichia phage vB_EcoM_PhAPEC2]; 1 TMD	0.0	Peptidase_U32[PF01136]; Peptidase family U32	1,00E-26
39	25024..25227	-	204	7.27	9.30	67	hypothetical protein				
40	25228..25788	-	561	21.63	5.39	186	hypothetical protein	hypothetical protein Acj61p078 [Acinetobacter phage Acj61]	1,00E-42		
41	25785..26138	-	354	13.30	5.86	117	hypothetical protein	hypothetical protein Acj9p083 [Acinetobacter phage Acj9]	2,00E-52		
42	26219..27820	-	1602	60.71	5.84	533	hypothetical protein	hypothetical protein EpJS10_0042 [Enterobacteria phage JS10]	0.0	NTP_transferase[PF00483]; Nucleotidyl transferase; APH[PF01636]; Phosphotransferase enzyme family	3.0e-07; 8.9e-06
43	27817..28269	-	453	17.15	9.39	150	hypothetical protein				
44	28325..31036	-	2712	104.26	5.76	903	DNA polymerase	DNA polymerase [Shigella phage Shf125875]	0.0	DNA_pol_B_exo1[PF03104]; DNA polymerase family B, exonuclease domain; DNA_pol_B[PF00136]; DNA polymerase family B	5.4e-23; 2.6e-43
45	31099..31326	-	228	8.75	4.73	75	hypothetical protein	hypothetical protein PM2_053 [Pectobacterium bacteriophage PM2]	3,00E-15		
46	31329..31694	-	366	14.48	8.50	121	translational repressor protein	translation repressor protein [Escherichia phage vB_EcoM_PhAPEC2]	3,00E-51	Translat_reg[PF01818]; Bacteriophage translational regulator	3.6e-60
47	31697..32269	-	573	22.42	5.77	190	clamp loader subunit, DNA polymerase accessory protein	clamp loader subunit DNA polymerase accessory protein [Shigella phage SP18]	4,00E-59		
48	32262..33224	-	963	36.08	8.15	320	clamp loader subunit, DNA polymerase accessory protein	clamp loader small subunit [Enterobacteria phage vB_EcoM_VR5]	1,00E-139	AAA[PF00004]; AAA proteins	1.5e-11
49	33239..33919	-	681	25.25	4.82	226	sliding clamp	sliding clamp, DNA polymerase accessory protein [Serratia phage PS2]	8,00E-71	DNA_PPF[PF02916]; DNA polymerase processivity factor; gp45-slide_C[PF09116; gp45 sliding clamp, C terminal	1.6e-28; 2.1e-37
50	33929..34243	-	315	11.96	9.47	104	RNA polymerase binding protein	RpbA RNA polymerase binding protein [Enterobacteria phage Phi1]	2,00E-21	Phage_RpbA[PF10789]; Phage RNA polymerase binding, RpbA	2.2e-28
51	34236..34418	-	183	7.38	4.57	60	hypothetical protein	gp45.2 conserved hypothetical protein [Enterobacteria phage RB69] gp45.2 conserved hypothetical protein [Enterobacteria phage RB69]	3,00E-11		

52	34415..36100	-	1686	64.08	8.57	561	recombination endonuclease subunit	recombination-related endonuclease [Salmonella phage STML-198]	0.0	AAA_23[PF13476]; AAA proteins	7.6e-18
53	36100..37116	-	1017	38.90	5.23	338	recombination endonuclease subunit	gp47 recombination endonuclease subunit [Enterobacteria phage T4]	2,00E-149	Metallophos_2[PF12850]; Calcineurin-like phosphoesterase superfamily domain	1.8e-11
54	37135..37320	-	186	7.15	9.48	61	hypothetical protein				
55	37298..37624	-	327	12.81	6.63	108	hypothetical protein	hypothetical protein PM2_066 [Pectobacterium bacteriophage PM2]	5,00E-13	DUF2654[PF10849]; Protein of unknown function (DUF2654)	1.9e-23
56	37813..38361	-	549	21.26	4.94	182	sigma factor for T4 late transcription	gp55 sigma factor [Acinetobacter phage Acj61]	3,00E-81		
57	38567..39988	-	1422	54.65	8.74	473	hypothetical protein	hypothetical protein PS2_067 [Serratia phage PS2]	0.0		
58	39988..40296	-	309	11.86	9.43	102	hypothetical protein	hypothetical protein [Salmonella phage S16]	2,00E-13		
59	40289..40567	-	279	10.55	5.26	92	glutaredoxin	NrdH glutaredoxin [Enterobacteria phage RB16]	6,00E-19		
60	40564..40806	-	243	9.32	4.41	80	hypothetical protein				
61	40799..41275	-	477	18.44	4.99	158	anaerobic NTP reductase, small subunit	NrdG anaerobic NTP reductase, small subunit [Enterobacteria phage T4]	3,00E-49	Fer4_12[PF13353]; 4Fe-4S single cluster domain; Radical_SAM[PF04055]; Radical SAM superfamily	1.6e-41; 1.5e-08
62	41313..41606	-	294	11.33	9.84	97	hypothetical protein	hypothetical protein SP18_gp070 [Shigella phage SP18]	2,00E-10		
63	41616..43460	-	1845	68.97	6.03	614	anaerobic NTP reductase, large subunit	NrdD anaerobic ribonucleotide reductase subunit [Klebsiella phage KP15]	0.0	NRDD[PF13597]; Anaerobic ribonucleoside-triphosphate reductase	7,00E-79
64	43457..43927	-	471	18.16	8.97	156	recombinase endonuclease VII	gp49 recombinase endonuclease VII [Enterobacteria phage RB14]	1,00E-70	Endonuclease_7[PF02945]; Recombination endonuclease VII; Endonuc-dimeris[PF09124]; T4 recombination endonuclease VII, dimerisation	8.1e-20; 4.2e-23
65	43970..44125	-	156	6.06	4.16	51	hypothetical protein			Glutaredoxin[PF00462]; Glutaredoxin	3.2e-10
66	44115..44387	-	273	10.48	8.56	90	thioredoxin	putative thioredoxin [Cronobacter phage S13]	2,00E-26		
67	44384..44653	-	270	10.24	5.15	89	hypothetical protein				
68	44662..44886	-	225	9.03	9.35	74	hypothetical membrane protein	2 TMD			
69	44889..45074	-	186	7.26	9.00	61	hypothetical protein	conserved hypothetical protein [Enterobacteria phage Bp7]	9,00E-15		
70	45077..46069	-	993	37.87	7.70	330	thioredoxin	thioredoxin [Salmonella phage STML-198]	5,00E-132	Nuc-transf[PF10127]; Predicted nucleotidyltransferase	1.5e-12
71	46069..46248	-	180	7.00	9.99	59	hypothetical membrane protein	1 TMD			

72	46245..46724	-	480	18.40	8.26	159	hypothetical protein	hypothetical protein RaK2_00242 [Enterobacteria phage vB_KleM-RaK2]	3,00E-16		
73	46741..46962	-	222	8.52	9.40	73	hypothetical protein	hypothetical protein CC31p101 [Enterobacteria phage CC31]	1,00E-05		
74	46970..47536	-	567	21.26	6.73	188	hypothetical protein				
75	47538..47705	-	168	6.39	4.65	55	hypothetical protein				
76	47738..48466	-	729	27.39	9.30	242	hypothetical protein	hypothetical protein CC2_405 [Aeromonas phage CC2]; 3 TMD	6,00E-08		
77	48466..48585	-	120	4.66	5.08	39	hypothetical protein				
77A	48582..48683	-	102	3.81	9.99	33	hypothetical protein				
78	48684..48890	-	207	8.08	9.51	68	hypothetical membrane protein	hypothetical protein SP101_00105 [Salmonella phage FSL SP-101]; 2 TMD	4,00E-09	Imm_superinfect[PF14373]; Superinfection immunity protein	3.6e-16
79	48890..49090	-	201	7.57	5.25	66	hypothetical membrane protein	1 TMD			
80	49096..49269	-	174	6.72	6.80	57	hypothetical protein				
81	49269..49391	-	123	4.87	10.08	40	hypothetical protein	hypothetical protein JS09_0195 [Escherichia phage vB_EcoM_JS09]	3,00E-09		
82	49393..49656	-	264	10.04	5.30	87	hypothetical protein	hypothetical protein PG7_107 [Enterobacter phage PG7]	6,00E-28	zf-dskA_traR[PF01258]; Prokaryotic dksA/traR C4-type zinc finger	1,00E-06
83	49653..49964	-	312	12.70	6.03	103	hypothetical protein				
84	49973..50137	-	165	6.37	9.46	54	hypothetical protein	hypothetical protein S13_136 [Cronobacter phage S13]	1,00E-13		
85	50201..50623	-	423	15.79	4.51	140	hypothetical protein				
86	50625..51227	-	603	23.22	6.75	200	hypothetical protein				
87	51326..51700	-	375	14.14	4.92	124	hypothetical protein	hypothetical protein PS2_107 [Serratia phage PS2]	2,00E-26		
88	51693..51929	-	237	9.02	4.91	78	lysis inhibition regulator	lysis inhibition regulator [Enterobacter phage PG7]	2,00E-15		
89	52014..52226	-	213	8.36	9.41	70	hypothetical protein				
90	52250..52816	-	567	21.15	5.96	188	thymidine kinase	thymidine kinase [Citrobacter phage Moon]	2,00E-67	TK[PF00265]; Thymidine kinase	1.3e-48
91	52818..53027	-	210	8.31	9.30	69	hypothetical protein				
91A	53024..53131	-	108	4.05	5.90	35	hypothetical membrane protein	1 TMD			
91B	53140..53349	-	210	8.19	8.96	69	hypothetical membrane protein	2 TMD			
92	53346..53786	-	441	16.65	9.32	146	hypothetical protein	hypothetical protein BN80_112 [Yersinia phage phiR1-RT]	9,00E-17		
93	53783..54097	-	315	11.60	5.09	104	valyl tRNA synthetase modifier	valyl tRNA synthetase modifier [Salmonella phage S16]	5,00E-19		

94	54101..54661	-	561	21.58	8.41	186	hypothetical protein	Vs.1 conserved hypothetical protein [Enterobacteria phage vB_EcoM-VR7]	4,00E-45	REGB_T4[PF10715]; Endoribonuclease RegB T4-bacteriophage encoded	6.2e-24
95	54658..55077	-	420	15.93	9.39	139	site-specific RNA endonuclease	site-specific RNA endonuclease [Salmonella phage S16]	5,00E-26	REGB_T4[PF10715]; Endoribonuclease RegB T4-bacteriophage encoded	7.1e-19
96	55077..55475	-	399	15.66	9.43	132	endonuclease V, N-glycosylase UV repair enzyme	endonuclease V, N-glycosylase UV repair enzyme [Enterobacter phage PG7]	3,00E-43	Pyr_excise[PF03013]; Pyrimidine dimer DNA glycosylase	1.8e-41
97	55528..55896	-	369	13.86	5.67	122	hypothetical protein	hypothetical protein RaK2_00521 [Enterobacteria phage vB_KleM-RaK2]	5,00E-42	Gly_radical[PF01228]; Glycine radical	2.1e-23
98	55896..56060	-	165	6.29	6.54	54	hypothetical protein				
99	56063..56554	-	492	19.10	8.83	163	hypothetical protein	hypothetical protein VR26_120 [Enterobacteria phage vB_EcoM_VR26]	8,00E-06		
100	56607..57065	-	459	17.69	8.78	152	hypothetical protein	hypothetical protein BN79_017 [Yersinia phage phiR201]	2,00E-25		
101	57085..57450	-	366	13.74	9.36	121	endolysin	endolysin [Yersinia phage PY100]	5,00E-46	Peptidase_M15_4[PF13539]; D-alanyl-D-alanine carboxypeptidase	7.2e-14
102	57490..57933	-	444	17.11	4.53	147	hypothetical protein				
103	57987..58391	-	405	15.46	5.55	134	nudix hydrolase	NudE nudix hydrolase [Klebsiella phage KP15]	6,00E-35	NUDIX[PF00293]; Nudix family	4.8e-18
104	58401..58622	-	222	8.44	4.28	73	hypothetical protein				
105	58699..58974	-	276	10.46	5.22	91	hypothetical membrane protein	2 TMD			
106	59054..59650	-	597	22.47	5.82	198	hypothetical protein	hypothetical protein Lw1_gp251 [Escherichia phage Lw1]	4,00E-32		
107	59647..59841	-	195	7.24	8.93	64	hypothetical protein				
108	59904..60176	-	273	10.52	4.57	90	hypothetical protein	hypothetical protein PM2_261 [Pectobacterium bacteriophage PM2]	2,00E-19		
109	60217..60699	-	483	18.60	8.36	160	hypothetical protein				
110	60692..61147	-	456	17.73	5.10	151	hypothetical protein				
111	61154..61444	-	291	10.73	8.48	96	hypothetical protein	hypothetical protein PhAPEC2_34 [Escherichia phage vB_EcoM_PhAPEC2]	3,00E-47		
112	61447..61692	-	246	9.25	9.61	81	hypothetical protein				
113	61791..62138	-	348	13.50	4.65	115	hypothetical protein				
114	62249..62653	-	405	14.31	6.02	134	hypothetical protein			YadA_anchor[PF03895]; YadA-like C-terminal region	6.3e-16
115	62662..63012	-	351	13.15	4.67	116	hypothetical protein			LysM[PF01476]; LysM domain	1.1e-05
116	63325..63645	-	321	12.27	4.13	106	hypothetical protein	hypothetical protein GAP32_374 [Cronobacter phage vB_CsaM_GAP32]	6,00E-21		
117	64015..64275	-	261	10.27	5.18	86	hypothetical protein				

118	64485..64667	-	183	6.73	9.30	60	hypothetical membrane protein	1 TMD			
119	64995..65267	-	273	10.84	4.23	90	hypothetical protein				
120	65314..65469	-	156	5.87	9.47	51	hypothetical protein	hypothetical protein CPT_Moon160 [Citrobacter phage Moon]	9,00E-16		
121	65813..65965	-	153	5.65	5.00	50	hypothetical protein				
122	66010..66393	-	384	14.31	7.64	127	hypothetical protein	hypothetical protein PY54p66 [Yersinia phage PY54]	3,00E-52		
123	66390..66743	-	354	13.46	6.83	117	hypothetical protein	hypothetical protein S13_183 [Cronobacter phage S13]	1,00E-09		
124	66961..67218	-	258	9.80	4.26	85	hypothetical protein				
124A	67220..67408		189	6.76	5.15	62	hypothetical protein				
125	67499..68020	-	522	20.50	9.41	173	hypothetical protein				
126	68077..68523	-	447	16.81	5.40	148	hypothetical protein	gp57B conserved hypothetical protein [Enterobacteria phage T4]	6,00E-59		
127	68513..68782	-	270	10.22	4.15	89	hypothetical protein				
128	68763..69422	-	660	25.62	7.08	219	dNMP kinase	gp1 dNMP kinase [Enterobacteria phage CC31]	8,00E-31		
129	69426..70004	-	579	21.79	4.94	192	tail completion and sheath stabilizer protein	gp3 tail completion and sheath stabilizer protein [Enterobacteria phage CC31]	8,00E-68	Phage_T4_gp19[PF06841]; T4-like virus tail tube protein gp19	4.3e-12
130	70211..71020	-	810	30.99	10.09	269	DNA end protector protein	DNA end protector protein [Serratia phage PS2]	1,00E-116		
131	71017..71466	-	450	17.84	9.86	149	head completion protein	gp4 head completion protein [Enterobacteria phage vB_EcoM-VR7]	2,00E-71		
132	71517..72068	+	552	21.53	5.04	183	baseplate wedge subunit	baseplate wedge subunit [Salmonella phage S16]	2,00E-85	Phage_gp53[PF11246]; Base plate wedge protein 53	1.1e-76
133	72065..73795	+	1731	64.09	5.36	576	baseplate hub subunit and tail lysozyme	baseplate hub subunit and tail lysozyme [Shigella phage SP18]	0.0	Gp5_OB[PF06714]; Gp5 N-terminal OB domain; Phage_lysozyme[PF00959]; Glycoside hydrolase family 24	1.3e-46; 1.5e-12
134	73795..75861	+	2067	78.14	4.93	688	hypothetical protein	hypothetical protein SP18_gp165 [Shigella phage SP18]	0.0		
135	75863..77782	+	1920	72.90	5.07	639	baseplate wedge subunit	gp6 base plate wedge [Enterobacteria phage CC31]	0.0		
136	77782..80889	+	3108	120.86	6.35	1035	baseplate wedge subunit	baseplate wedge subunit [Enterobacteria phage vB_EcoM_VR26]	0.0		
137	80889..81896	+	1008	38.35	4.81	335	baseplate wedge tail fiber connector	gp8 base plate wedge [Enterobacteria phage CC31]	1,00E-163	Phage-Gp8[PF09215]; Bacteriophage T4, Gp8	1.6e-138
138	81955..82806	+	852	31.07	5.91	283	baseplate wedge subunit and tail pin	gp9 base plate wedge component [Aeromonas phage 25]	3,00E-74	T4_gp9_10[PF07880]; Bacteriophage T4 gp9/10-like protein	2.3e-67

139	82803..84029	+	1227	45.75	5.46	408	baseplate wedge subunit and tail pin	baseplate wedge subunit and tail pin [Cronobacter phage vB_CsaM_GAP161]	1,00E-116	T4_gp9_10[PF07880]; Bacteriophage T4 gp9/10-like protein	8.3e-75
140	84146..84538	+	393	14.17	4.93	130	baseplate wedge subunit and tail pin	putative baseplate wedge subunit and tail pin [Escherichia phage HY01]	1,00E-42		
141	84538..85158	+	621	22.62	4.71	206	baseplate wedge subunit and tail pin	gp11 baseplate wedge completion tail pin [Acinetobacter phage 133]	3,00E-28	GP11[PF08677]; GP11 baseplate wedge protein	4.7e-43
142	85158..86501	+	1344	4.62	9.40	447	short tail fibers protein	putative short tail fibers [Cronobacter phage S13]	3,00E-71		
143	86511..88247	+	1737	64.05	4.59	578	fibritin neck whiskers	fibritin neck whiskers [Pectobacterium bacteriophage PM2]	2,00E-79	Fibritin_C[PF07921]; Fibritin C-terminal region	7.1e-15
144	88284..89210	+	927	34.43	4.53	308	neck protein	gp13 neck protein [Enterobacteria phage T4]	5,00E-154		
145	89214..89957	+	744	28.56	4.42	247	neck protein	gp14 neck protein [Enterobacteria phage JS10]	2,00E-123	T4_neck-protein[PF11649]; Virus neck protein	1.9e-94
146	89980..90813	+	834	32.48	5.35	277	tail sheath stabilizer and completion protein	gp15 tail sheath stabilizer and completion protein [Enterobacteria phage JS10]	6,00E-118		
147	90816..91295	+	480	18.05	4.70	159	terminase, small subunit	terminase DNA packaging enzyme, small subunit [Escherichia phage vB_EcoM_PhAPEC2]	2,00E-63	DNA_Packaging[PF11053]; Terminase DNA packaging enzyme	3.5e-60
148	91267..93066	+	1800	68.38	5.26	599	terminase, large subunit	gp17 terminase DNA packaging enzyme, large subunit [Enterobacteria phage CC31]	0.0	Terminase_6[PF03237]; Terminase-like family	6.2e-78
149	93096..95090	+	1995	72.50	5.27	664	tail sheath protein	gp18 tail sheath protein [Enterobacteria phage JS98]	0.0	Phage_sheath_1[PF04984]; Phage tail sheath protein	1.3e-144
150	95127..95618	+	492	18.67	4.97	163	tail tube protein	gp19 tail tube protein [Enterobacteria phage JS98]	4,00E-91	Phage_T4_gp19[PF06841]; T4-like virus tail tube protein gp19	1.6e-12
151	95676..97226	+	1551	60.08	5.41	516	portal vertex protein of head	portal vertex of the head [Salmonella phage STML-198]	0.0	Peptidase_S80[PF07230]; Bacteriophage T4-like capsid assembly protein (Gp20)	9.1e-223
152	97227..97448	+	222	8.45	4.54	73	prohead core protein	prohead core protein [Acinetobacter phage ZZ1]	8,00E-06		
153	97461..97883	+	423	16.01	10.09	140	prohead core protein	prohead core protein [Shigella phage SP18]	6,00E-49		
154	97880..98506	+	627	22.91	5.62	208	prohead core scaffold protein and protease	prohead core scaffolding protein and protease [Salmonella phage S16]	3,00E-102	Peptidase_U9[PF03420]; Prohead core protein protease	1.4e-91
155	98539..99330	+	792	29.53	4.49	263	prohead core scaffold protein	gp22 prohead core scaffold protein [Acinetobacter phage Acj61]	3,00E-70		
156	99351..100901	+	1551	56.62	5.10	516	major capsid protein	major capsid protein [Citrobacter phage Moon]	0.0	Gp26[PF07068]; Major capsid protein Gp23	1.5e-259
157	100995..102245	+	1251	45.99	4.73	416	head vertex protein	phage capsid vertex [Yersinia phage phiR1-RT]	7,00E-147	Gp26[PF07068]; Major capsid protein Gp23	4,00E-10
158	102304..102867	-	564	21.24	7.67	187	hypothetical protein	hypothetical protein PS2_186 [Serratia phage PS2]	5,00E-40		

159	102900..103076	-	177	6.57	10.30	58	hypothetical membrane protein	Hypothetical-Protein | belonging to T4-LIKE GC: 762 [Synechococcus phage S-PM2]; 2 TMD	6,00E-08		
160	103078..103323	-	246	9.24	5.73	81	hypothetical protein				
161	103317..104318	-	1002	38.09	5.97	333	RNA ligase 2	RNA ligase 2 [Escherichia phage wV7]	1,00E-79	RNA_ligase[PF09414]; RNA ligase	1.7e-38
162	104320..104667	-	348	13.37	5.66	115	hypothetical protein				
163	104677..104961	-	285	11.15	9.52	94	hypothetical protein				
164	104958..105149	-	192	7.64	10.13	63	hypothetical protein	gp24.3 hypothetical protein [Enterobacteria phage JS10]	3,00E-17	DUF2774[PF11242]; Protein of unknown function (DUF2774)	1.4e-20
165	105136..105309	-	174	6.73	4.57	57	hypothetical protein				
166	105312..105452	-	141	5.23	4.94	46	hypothetical protein				
167	105484..105759	+	276	10.54	9.65	91	hypothetical membrane protein	1 TMD			
168	105743..106165	-	423	16.51	5.65	140	hypothetical protein				
169	106175..106408	-	234	9.07	6.08	77	hypothetical protein				
170	106431..106652	-	222	8.56	5.27	73	hypothetical protein				
171	106810..107169	+	360	13.61	9.18	119	hypothetical protein	hypothetical protein [Escherichia phage ECML-4]	4,00E-10		
172	107199..108113	-	915	33.28	4.83	304	baseplate tail tube initiator	gp54 base plate tail tube initiator [Enterobacteria phage CC31]	5,00E-160	Phage_T4_gp19[PF06841]; T4-like virus tail tube protein gp19	2.3e-11
173	108113..109156	-	1044	37.94	6.00	347	baseplate tail tube cap	gp48 base plate tail tube cap [Enterobacteria phage CC31]	2,00E-153	T4_tail_cap[PF11091]; Tail-tube assembly protein	6.8e-139
174	109156..110868	-	1713	63.22	4.83	570	baseplate hub subunit, tail length determinator	baseplate hub subunit tail length determinator [Escherichia phage wV7]; 1 TMD	9,00E-59		
175	110855..111349	-	495	18.59	5.32	164	baseplate hub distal subunit	baseplate hub distal subunit [Salmonella phage S16]	3,00E-30	Phage_hub_GP28[PF11110]; Baseplate hub distal subunit	3.1e-33
176	111336..112481	-	1146	43.73	5.11	381	baseplate hub subunit	baseplate hub subunit [Enterobacteria phage Bp7]	1,00E-164	Phage-tail_1[PF09097]; Baseplate structural protein, domain 1; Phage-tail_2[PF09096]; Baseplate structural protein, domain 2	3.1e-33; 6.7e-73
177	112459..113232	-	774	30.38	5.33	257	baseplate hub assembly protein	gp51 baseplate hub assembly protein [Enterobacteria phage vB_EcoM-VR7]	9,00E-67	T4_baseplate[PF12322]; T4 bacteriophage base plate protein	3.4e-43
178	113278..113862	+	585	22.46	4.59	194	baseplate hub subunit	base plate hub subunit [Enterobacter phage PG7]	2,00E-25	T4_baseplate[PF12322]; T4 bacteriophage base plate protein	7.1e-29
179	113855..114241	+	387	14.55	5.40	128	baseplate wedge subunit	gp25 baseplate wedge subunit [Acinetobacter phage 133]	1,00E-58	GPW_gp25[PF04965]; Gene 25-like lysozyme	1.9e-16
180	114485..114895	+	411	15.66	7.70	136	recombination, repair and ssDNA binding protein	recombination, repair and ssDNA binding protein [Salmonella phage S16]	1,00E-50	UvsY[PF11056]; Recombination, repair and ssDNA binding protein UvsY	1.7e-46

181	114892..115272	+	381	14.14	4.63	126	hypothetical protein	UvsY.-2 conserved hypothetical protein [Enterobacteria phage T4]	4,00E-12	DUF2685[PF10886]; Protein of unknown function (DUF2685)	1.7e-15
182	115288..115509	-	222	8.61	4.60	73	hypothetical protein	hypothetical protein RB32ORF181w [Enterobacteria phage RB32]	2,00E-22	UvsW[PF11637]; ATP-dependant DNA helicase UvsW	6.8e-23
183	115506..117008	-	1503	57.04	9.00	500	RNA-DNA and DNA-DNA helicase, ATPase	UvsW RNA-DNA and DNA-DNA helicase ATPase [Enterobacteria phage JS98]	0.0	ResIII[PF04851]; Type III restriction enzyme, res subunit; Helicase_C[PF00271]; Helicase conserved C-terminal domain	1.9e-11; 5.9e-05
184	117065..117775	+	711	27.50	4.54	236	inhibitor of prohead protease	inhibitor of prohead protease gp21 [Enterobacteria phage CC31]	6,00E-50		
185	117788..118867	+	1080	39.98	4.57	359	head outer capsid protein	head outer capsid protein [Acinetobacter phage ZZ1]	3,00E-59		
186	118901..119167	-	267	9.89	4.38	88	hypothetical protein				
187	119227..119430	-	204	8.10	4.28	67	hypothetical protein				
188	119494..121536	-	2043	77.06	5.58	680	RNA polymerase ADP-ribosylase	Alt RNA polymerase ADP-ribosylase [Enterobacteria phage JS10]	8,00E-143	ADPrib_exo_Tox[PF03496]; ADP-ribosyltransferase exoenzyme	4.2e-24
189	121614..121853	-	240	9.02	4.48	79	hypothetical protein				
190	121931..122056	-	126	4.79	6.09	41	hypothetical protein				
191	122064..123530	-	1467	55.62	5.84	488	DNA ligase	gp30 DNA ligase [Acinetobacter phage Acj61]	3,00E-128	DNA_ligase_A_M[PF01068]; ATP dependent DNA ligase domain	5.4e-27
192	123540..124175	-	636	24.27	5.22	211	hypothetical protein	hypothetical protein PS2_213 [Serratia phage PS2]	3,00E-79		
193	124172..124627	-	456	17.52	4.56	151	hypothetical protein				
194	124637..125083	-	447	16.98	4.81	148	hypothetical protein				
195	125128..125583	-	456	17.43	9.68	151	hypothetical protein	gp30.3 conserved hypothetical protein [Acinetobacter phage Ac42]	3,00E-42	Phage_30_3[PF08010]; Bacteriophage protein GP30.3	3.6e-47
196	125564..126055	-	492	18.92	4.68	163	hypothetical protein				
197	126167..126403	-	237	9.14	7.89	78	hypothetical protein				
198	126509..126643	-	135	5.12	11.17	44	hypothetical protein				
199	126653..126898	-	246	9.26	9.35	81	rIII lysis inhibition accessory protein, rapid lysis phenotype	rIII lysis inhibition accessory protein, rapid lysis phenotype [Serratia phage PS2]	9,00E-31		
200	126971..127294	-	324	11.63	6.73	107	head assembly cochaperone with GroEL	head assembly co-chaperonin [Citrobacter phage Moon]	8,00E-30	Cpn10[PF00166]; Chaperonin 10 Kd subunit	3.2e-12
201	127294..127611	-	318	12.42	8.69	105	hypothetical protein	hypothetical protein [Salmonella phage STML-198]	9,00E-16	DUF2693[PF10902]; Protein of unknown function (DUF2693)	3.8e-16
202	127608..128162	-	555	20.26	7.64	184	dCMP deaminase	dCMP deaminase [Yersinia phage phiR1-RT]	6,00E-86	dCMP_cyt_deam_1[PF00383]; Cytidine and deoxycytidylate deaminase zinc-binding region	3.7e-32

203	128162..128446	-	285	11.40	5.47	94	hypothetical protein				
204	128446..128721	-	276	10.52	6.55	91	hypothetical protein				
205	128721..129683	-	963	35.52	6.80	320	phospho-2-dehydro-3-deoxyheptonate aldolase	phospho-2-dehydro-3-deoxyheptonate aldolase [Escherichia phage vB_EcoM_JS09]	3,00E-125	DAHP_synth_1[PF00793]; DAHP synthetase I family	3.8e-72
206	129673..129924	-	252	9.75	5.47	83	hypothetical protein				
207	129917..130126	-	210	7.88	5.65	69	hypothetical protein				
208	130135..130329	-	195	7.53	9.26	64	hypothetical protein				
209	130329..131327	-	999	39.27	6.53	332	3'-phosphatase, 5'-polynucleotide kinase	3'-phosphatase, 5'-polynucleotide kinase [Salmonella phage STML-198]	2,00E-79	AAA_33[PF13671]; AAA proteins	1.1e-17
210	131337..131516	-	180	7.01	4.61	59	hypothetical protein				
211	131513..132217	-	705	26.67	6.45	234	hypothetical protein				
212	132207..132503	-	297	11.35	7.68	98	hypothetical protein	PseT.2 conserved hypothetical protein [Enterobacteria phage JS10]	4,00E-20		
213	132500..132838	-	339	12.88	6.11	112	hypothetical protein	putative membrane protein [Enterobacter phage PG7]	5,00E-09	HN[PF00423]; Glycoside hydrolase family 83	6.1e-05
214	132853..133353	-	501	19.10	7.01	166	inhibitor of host transcription	inhibitor of host transcription [Salmonella phage S16]	4,00E-63		
215	133411..134529	-	1119	42.94	4.88	372	RNA ligase 1 and tail fiber attachment catalyst	putative RNA ligase 1 and tail fiber attachment catalyst [Cronobacter phage S13]	5,00E-113	RNA_lig_T4_1[PF09511]; RNA ligase	7.2e-39
216	134526..134936	-	411	16.20	8.89	136	endonuclease II	endonuclease II [Serratia phage PS2]	8,00E-52	GIY-YIG[PF01541]; GIY-YIG catalytic domain	1.8e-12
217	134964..136133	-	1170	45.58	5.02	389	aerobic NDP reductase, small subunit	ribonucleotide reductase B subunit [Enterobacteria phage RB32]	1,00E-172	Ribonuc_red_sm[PF00268]; Ribonucleotide reductase, small chain; Ribonuc_red_sm[PF00268]; Ribonucleotide reductase, small chain	8.1e-11; 5.9e-12
218	136135..136290	-	156	6.00	9.85	51	hypothetical protein				
219	136271..138508	-	2238	84.76	5.46	745	aerobic NDP reductase, large subunit	ribonucleotide reductase alpha subunit [Pectobacterium bacteriophage PM2]	0.0	ATP-cone[PF03477]; ATP cone domain; Ribonuc_red_lgN[PF00317]; Ribonucleotide reductase, all-alpha domain; Ribonuc_red_lgC[PF02867]; Ribonucleotide reductase, barrel domain	1.7e-12; 6.2e-12; 7.9e-127
220	138520..139113	-	594	23.27	8.69	197	hypothetical protein				
221	139094..139375	-	282	10.66	9.30	93	hypothetical protein	hypothetical protein VR20_242 [Enterobacteria phage vB_EcoM_VR20]	8,00E-44	DUF4326[PF14216]; Domain of unknown function (DUF4326)	5.8e-22
222	139534..139869	-	336	12.89	4.38	111	hypothetical protein				

223	139878..140720	-	843	32.58	5.74	280	thymidylate synthase	thymidylate synthase [Vibrio phage SSP002]	8,00E-137	Thymidylat_synt[PF00303]; Thymidylate synthase	7.3e-66
224	140705..140977	-	273	10.36	5.37	90	hypothetical protein				
225	140952..141359	-	408	15.63	8.48	135	hypothetical protein				
226	141352..141753	-	402	15.74	8.23	133	hypothetical protein				
227	141750..142334	-	585	22.59	6.83	194	dihydrofolate reductase	dihydrofolate reductase [Enterobacteria phage JSE]	2,00E-20	DHFR_1[PF00186]; Dihydrofolate reductase	3.8e-14
228	142437..143333	-	897	33.38	4.86	298	single stranded DNA-binding protein	gp32 single-stranded DNA binding protein [Enterobacteria phage CC31]	2,00E-127	gp32[PF08804]; gp32 DNA binding protein like	8.9e-43
229	143374..144027	-	654	25.68	9.08	217	DNA helicase loading protein	helicase loading protein [Enterobacteria phage vB_EcoM_VR20]	5,00E-115	T4_Gp59_N[PF08993]; T4 gene Gp59 loader of gp41 DNA helicase; T4_Gp59_C[PF08994]; T4 gene Gp59 loader of gp41 DNA helicase C-term	1.3e-43; 1.4e-40
230	144032..144301	-	270	10.05	4.55	89	late promoter transcription accessory protein	late promoter transcription accessory protein [Aeromonas phage Aes508]	3,00E-19		
231	144276..144545	-	270	10.40	5.04	89	dsDNA binding protein	DsbA dsDNA binding protein, late transcription [Enterobacteria phage T4]	2,00E-29	Phage_DsbA[PF11126]; Transcriptional regulator DsbA	3.1e-30
232	144554..145471	-	918	35.80	5.84	305	RNase H	ribonuclease [Serratia phage PS2]	3,00E-107	5_3_exonuc_N[PF02739]; 5'-3' exonuclease, N-terminal resolvase-like domain; RNaseH_C[PF09293]; T4 RNase H, C terminal	5.9e-09; 2.5e-43
233	145510..149166	+	3657	135.99	5.24	1218	long tail fiber, proximal subunit	long tail fiber proximal subunit [Salmonella phage STML-198]	0.0		
234	149159..150277	+	1119	42.12	4.92	372	long tail fiber, proximal connector	long tail fiber, proximal connector [Enterobacteria phage Bp7]	9,00E-39		
235	150479..150946	+	468	16.85	4.76	155	long tail fiber, distal connector	long tail fiber, distal connector [Enterobacteria phage Bp7]	2,00E-17	Phage_T4_gp36[PF03903]; Phage T4 tail fibre	3.7e-28
236	150946..153201	+	2256	82.30	9.11	751	long tail fiber distal subunit	long tail fiber distal subunit [Escherichia phage e11/2]	3,00E-18	Peptidase_S74[PF13884]; Chaperone of endosialidase	1.7e-09
237	153182..153697	+	516	19.22	9.00	171	distal long tail fiber assembly catalyst	distal long tail fiber assembly catalyst [Klebsiella phage KP27]	3,00E-12	GP38[PF05268]; Phage tail fibre adhesin Gp38	2,00E-12
238	153707..154330	+	624	24.24	5.87	207	holin lysis mediator	holin lysis mediator [Enterobacteria phage CC31]; 1 TMD	9,00E-65	Phage_holin_T[PF11031]; Bacteriophage T holin	9.9e-70
239	154333..154767	-	435	16.32	8.68	144	hypothetical protein				
240	154770..155027	-	258	9.64	5.47	85	hypothetical protein				
241	155027..155404	-	378	14.61	4.81	125	hypothetical protein				
242	155401..155916	-	516	19.71	4.75	171	hypothetical protein				
243	155918..156190	-	273	10.38	4.98	90	anti-sigma 70 protein	anti-sigma 70 protein [Enterobacteria phage CC31]	3,00E-19	AsiA[PF09010]; Anti-Sigma Factor A;	4.7e-25

244	156165..156413	-	249	9.39	6.07	82	hypothetical protein				
245	156420..157025	-	606	22.63	9.28	201	activator of middle transcription	activator of middle transcription [Enterobacteria phage vB_EcoM_VR5]	9,00E-55	MotA_activ[PF09114]; Transcription factor MotA, activation domain; MotCF[PF09158]; Bacteriophage T4 MotA, C-terminal	2E-30; 8.1e-26
246	157154..157591	-	438	17.25	6.29	145	hypothetical protein				
247	157588..157779	-	192	7.83	5.02	63	hypothetical protein	gp15 [Erwinia phage vB_EamM-Y2]	2,00E-05		
248	157779..158057	-	279	10.63	5.56	92	hypothetical protein				
249	158136..159494	-	1359	51.81	6.75	452	DNA topoisomerase	DNA topisomerase II medium subunit [Salmonella phage S16]	0.0	DNA_topoisoIV[PF00521]; DNA gyrase/topoisomerase IV, subunit A	2.4e-91
250	159549..160040	-	492	18.95	9.78	163	nucleoid disruption protein	Ndd nucleoid disruption protein [Acinetobacter phage Acj9]	9,00E-08	Phage_T4_Ndd[PF06591]; T4-like phage nuclear disruption protein (Ndd)	1,00E-08
251	160082..160702	-	621	23.56	8.22	206	DNA endonuclease IV	endonuclease IV [Enterobacteria phage RB51]	7,00E-55		
252	160752..161819	-	1068	40.12	8.77	355	protector from prophage-induced early lysis	protector from prophage-induced early lysis [Pectobacterium bacteriophage PM2]	2,00E-101		
