# Supplementary material for: Development of a Phage Cocktail to Control Proteus mirabilis Catheter-associated Urinary Tract Infections
Source: Front Microbiol. 2016 Jun 28;7:1024. doi: 10.3389/fmicb.2016.01024 (PMC4923195; doi:10.3389/fmicb.2016.01024)
Supplement: Supplementary file 2 [file Image_1.PDF]

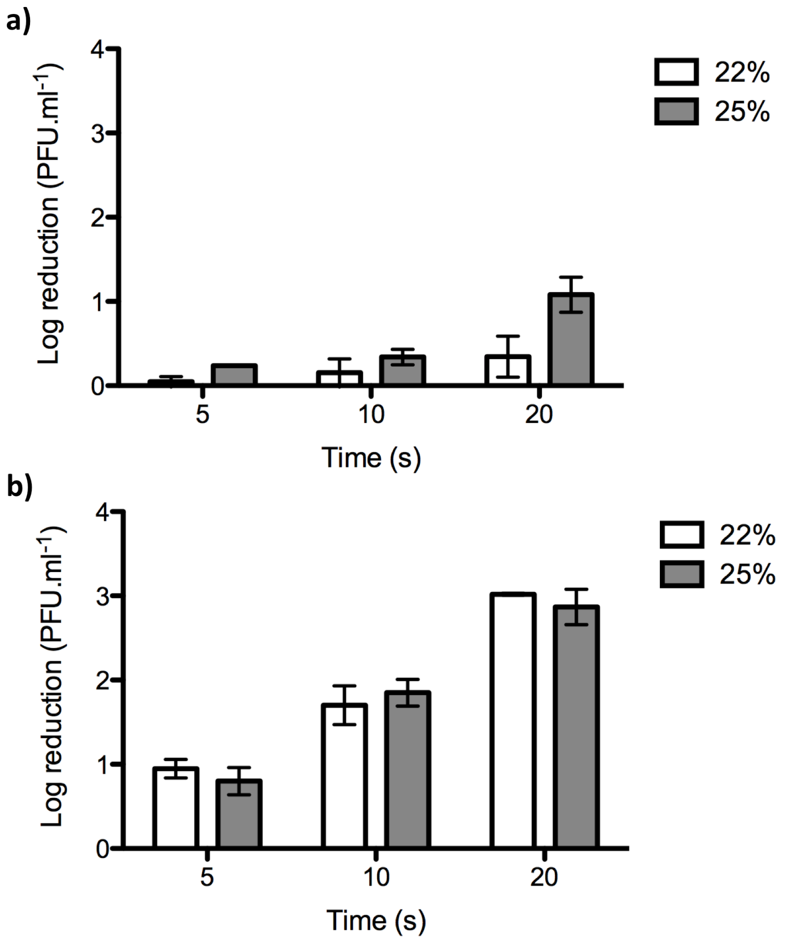


Fig. S1 *P. mirabilis* phages resistance to sonication under different amplitudes and time of exposure:  a) Pm5460; b) Pm5461. Data points represent an average of three independent experiments and error bars indicate standard error of means.
