# Supplementary material for: Development of a Phage Cocktail to Control Proteus mirabilis Catheter-associated Urinary Tract Infections
Source: Front Microbiol. 2016 Jun 28;7:1024. doi: 10.3389/fmicb.2016.01024 (PMC4923195; doi:10.3389/fmicb.2016.01024)
Supplement: Supplementary file 3 [file Image_2.PDF]

a)

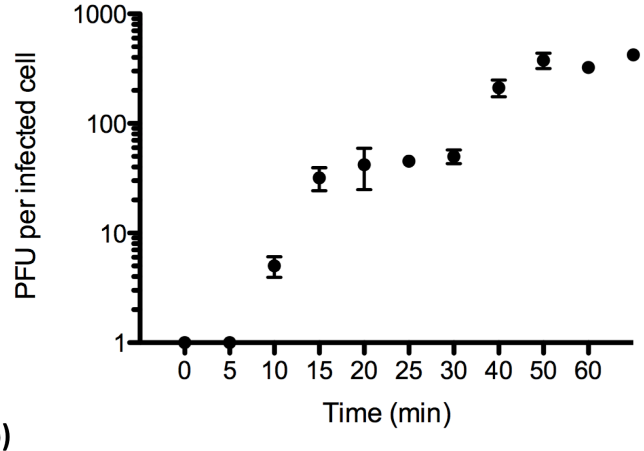

b)

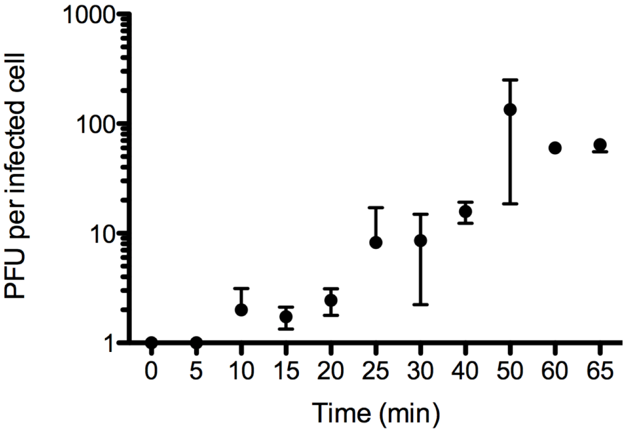

Fig. S2 – One-step growth curves of *P. mirabilis* phages Pm5460 (a) and Pm5461 (b). Data points represent an average of three independent experiments and error bars indicate standard error of means.
