# Supplementary material for: Development of a Phage Cocktail to Control Proteus mirabilis Catheter-associated Urinary Tract Infections
Source: Front Microbiol. 2016 Jun 28;7:1024. doi: 10.3389/fmicb.2016.01024 (PMC4923195; doi:10.3389/fmicb.2016.01024)
Supplement: Supplementary file 4 [file Image_3.PDF]

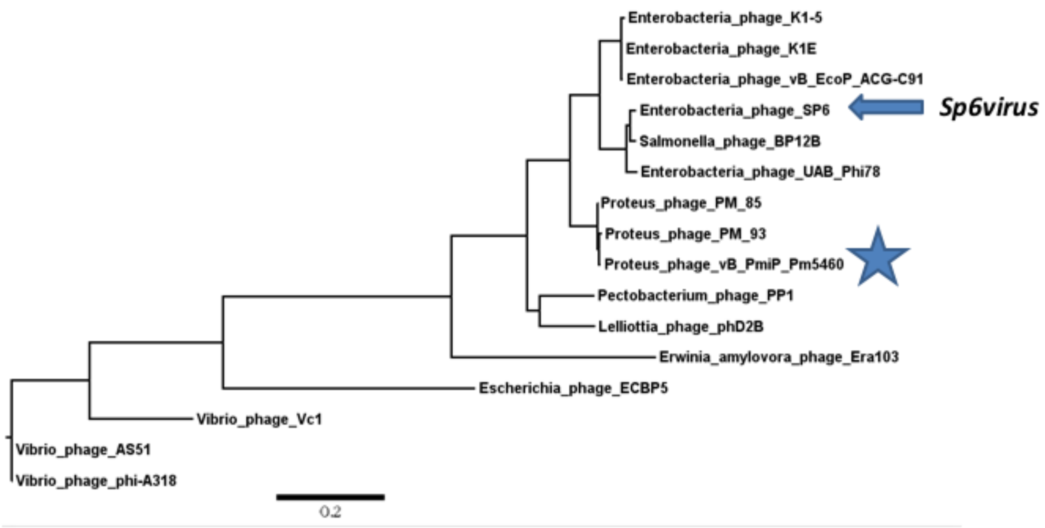

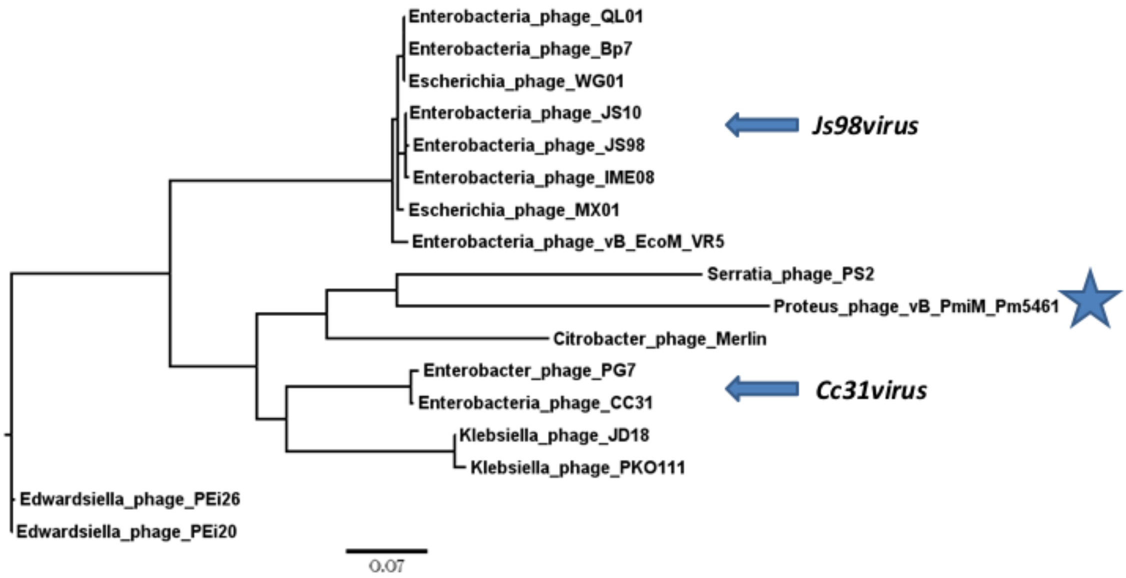

Fig. S3 - Phylogenetic analysis using homologous proteins identified using BLASTP against the virus database at NCBI. A) DNA polymerase of 5460; B) Large subunit terminase of 5461.The trees were generated using the “one click” mode at phylogeny.fr. The ICTV approved taxa are indicated with arrows and the *Proteus* phages described in this paper are indicated with a star.
